# Bioactive Compounds in Fruit Wines: A Narrative Review

**DOI:** 10.3390/foods15193505

**Published:** 2026-09-30

**Authors:** Uroš Čakar, Tijana Ilić, José Yamil Neira Hinojosa, Carlos L. Cespedes Acuña, Miona Belović

**Affiliations:** 1 Department of Bromatology, Faculty of Pharmacy, University of Belgrade, Vojvode Stepe 450, 11221 Belgrade, Serbia; tijana.ilic@pharmacy.bg.ac.rs; 2 Department of Instrumental Analysis, Faculty of Pharmacy, Universidad de Concepción, Concepción 4070409, Chile; yneira@udec.cl; 3 Departamento de Ciencias Basicas, Facultad de Ciencias, Universidad del Bio Bio, Av. Andres Bello #720, Chillan 3810189, Chile; ccespedes@ubiobio.cl; 4 Institute of Food Technology, University of Novi Sad, Bulevar cara Lazara 1, 21000 Novi Sad, Serbia

**Keywords:** fruit wine, fermentation, bioactive compounds, phenolic compounds, antioxidant activity, functional food, fruit pomace, circular economy

## Abstract

Fruit wines produced from non-grape fruits are gaining increasing attention as value-added fermented beverages due to their diverse chemical composition and content of bioactive compounds. This narrative review provides a comparative overview of fruit wines obtained from berries, drupes, pome fruits, citrus fruits, and tropical fruits, with particular emphasis on their physicochemical characteristics, phenolic composition, antioxidant properties, and technological aspects of production. Rather than considering fruit wines as a single category, the available evidence is examined across different fruit groups to identify characteristic patterns in the occurrence and relative abundance of bioactive compounds and their associated antioxidant activity. Berry wines are generally distinguished by their high phenolic and anthocyanin contents, whereas wines produced from other fruit categories exhibit distinct profiles of phenolic acids, flavonoids, and other bioactive constituents. Differences in raw materials and technological conditions, including fermentation and processing strategies, contribute substantially to the variability observed in the composition and functional properties of the resulting wines. The review also considers fruit-wine pomace as a potentially valuable source of residual bioactive compounds, highlighting opportunities for its valorization within sustainable and circular production systems. However, differences in analytical methods, reporting units, sample matrices, and experimental conditions currently limit direct quantitative comparisons among studies. Overall, this review integrates compositional, technological, and antioxidant-activity data across major fruit categories, identifies current knowledge gaps, and highlights opportunities for improving fruit-wine production, analytical characterization, and the sustainable utilization of fruit-processing by-products.

## 1. Introduction

As a cultural phenomenon, wine has held a significant place throughout history, as evidenced by the fact that the technology for processing grapes and other fruit into wine is among the oldest production processes still used today. This is confirmed by traces of wine production in the Caucasus Mountains dating back to 6000 BC, in ancient Egypt and Sumeria from the 3rd millennium BC, and in India from 1500 BC, where the text by the Chakra Samhita highlights the positive effects of wine on the human body, as well as its ability to create a sense of satisfaction [1,2]. Today, wine is produced on every continent except Antarctica, and the most commercially important wines are those made from grapes, while the production of fruit wines is affected by various factors, the most significant being geographical location and climatic conditions in a particular region of the world. However, it is important to note that the term “wine” is used exclusively for alcoholic beverages produced from grapes, while fruit wines can be defined as alcoholic beverages obtained by fermenting the juice or pulp of any fruit other than grapes [3,4,5].

The quantities of fruits other than grapes produced worldwide are increasing every year. Serbia contributes significantly to global and European fruit production statistics as a leading producer of raspberries and blackberries, as well as stone fruits such as plums. In the Southern Hemisphere, Chile is considered one of the largest producers of blueberries, stone fruits (cherries, plums, and peaches), and apples [6]. It is also important to highlight other major fruit-producing countries, including China, India, Brazil, the USA, Italy, Mexico, South Africa, and New Zealand [7]. Part of this large volume of fruit is consumed fresh, while the rest is processed, which extends its shelf life and availability worldwide throughout the year. One such process is fermentation, which when applied to fruit processing, produces wines that, in terms of nutritional properties and content of naturally occurring bioactive compounds, are in no way inferior to those produced from grapes [8,9,10].

The process of producing fruit wines is similar to that used for white and red grape wines. Therefore, there is no obstacle to producing fruit wines in facilities designed for grape processing [11]. However, the production of fruit wines is limited to countries or regions where specific fruits are grown and available in quantities sufficient for winemaking. Through partial or complete alcoholic fermentation, malolactic fermentation, and other technological winemaking processes, high-quality fruit wines can be produced. Nevertheless, compared with the volume of grape wine currently produced and consumed worldwide, the production and consumption of fruit wine remain significantly lower [12,13]. Among the fruit wines, those made from apple (cider) and pear (perry) stand out as the most widely produced, though they are consumed in different parts of the world [14]. In regions of Europe, Asia, and North America where climatic conditions allow, wines are also produced from berries (blueberries, strawberries, raspberries) [15,16,17], citrus [18], and drupe fruits [19,20]. In addition to these groups, wines made from various tropical fruits that are indigenous to different parts of South America and Asia are also noteworthy [21,22,23,24].

The processes involved in wine production create a final product with added value compared to the raw material. Fruit wine is not just a mixture of ethanol and water, but a complex matrix that contains various biologically active compounds [25]. Phenolic and other bioactive compounds found in the solid parts of the fruit, such as the skin and seeds, are extracted into the aqueous ethanol fraction during winemaking. In this way, active compounds from plant tissues that are otherwise insoluble become more available for absorption after consuming fruit wine [26]. Wine production does not involve distillation, which is another reason why its nutrient content is richer than that of many other high-alcohol beverages. In spirits, the technological production process leads to the degradation of thermo-labile compounds, such as polyphenols and vitamins, which have beneficial health effects on the human body [12]. Biologically active compounds contribute to the potential therapeutic properties of fruits and products obtained from their processing, suggesting that such products may contribute to a healthy diet. This is why wine, whether made from grapes or other fruits, has an important position in a properly balanced diet.

The manufacture of fruit wines generates a massive quantity of by-products, which can be divided into several categories: waste from the processing of fruit varieties, fruit- and wine-processing wastewaters, lees (fermentation sediment rich in yeast biomass), and solid waste like pomace, peels, seeds, and stems [27]. Fruit wine pomace has a composition similar to other fruit wastes, especially to pomaces that are by-products of juice production. They are generally composed of skin, pulp, stems, and seeds and represent a rich source of pectin and other dietary fiber, phenolic compounds, and vitamins [28,29]. They can exert antioxidant, anti-carcinogenic, anti-diabetic, and anti-hypertensive activities [29]. Therefore, fruit wine and pomace obtained after the vinification procedure can be used to achieve the third Sustainable Development Goal (SDG3) established by the United Nations—good health and well-being. The presented paper is a narrative review, synthesizing existing literature on fruit wine and pomace composition, bioactivity, and health effects. It examines the technological processes used in fruit wine production, their quality parameters, and phenolic and antioxidant profiles. It also aims to evaluate the beneficial health effects of these bioactive compounds, including their role in reducing oxidative stress and preventing chronic non-communicable diseases such as cardiovascular disease, cancer, and hyperglycemia. Additionally, it highlights fruit wine pomace as an underutilized, rich source of bioactive compounds that can be valorized in line with circular economy principles and sustainable development goals.

## 2. Materials and Methods

### Literature Search

The preliminary literature search for this narrative review was conducted using the keywords “fruit wine” and “fruit wine pomace”. The individual fruits were identified during the preliminary search (e.g., blueberry, blackberry, raspberry, sour cherry, plum, apple, kiwi, dragon fruit, among others). Afterward, the names of individual fruits were combined with the keywords “physicochemical properties”, “phenolic compounds”, “antioxidant capacity”, and “bioactivity”. Searches were performed across the Scopus, PubMed, and Google Scholar databases, and retrieved articles were screened for relevance based on their focus on fruit wine composition, bioactive compound profiles, and biological activities. Given the narrative nature of this review, no strict systematic search protocol, predefined inclusion/exclusion criteria, or formal quality assessment tools were applied.

## 3. Fruit Wine Production

### 3.1. Production of Fruit Around the World and the Significance of Fruit Processing

**Figure 1:**
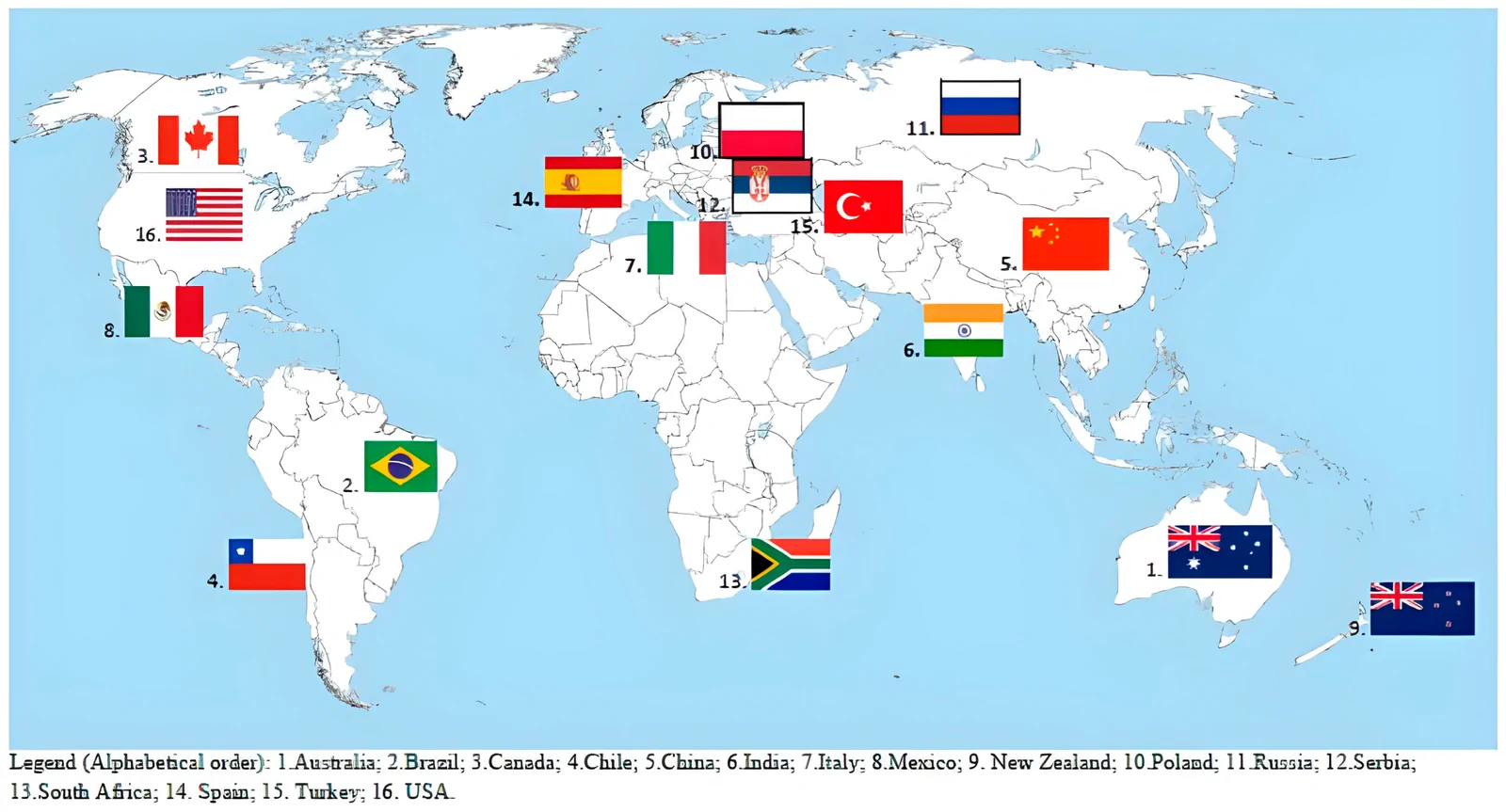
Some of the world’s leading fruit-producing countries.

Fruit production is one of the most important sectors of global agriculture, contributing significantly to food security, rural development, international trade, and agro-industrial growth. According to the Food and Agriculture Organization (FAO), global fruit production has increased considerably over recent decades, driven by rising consumer demand for fresh fruits, processed products, and functional foods. Major fruit-producing regions include Asia, Europe, Latin America, Africa, and North America, with China, India, Brazil, the United States, and Mediterranean countries (Figure 1) being among the leading producers of apples, grapes, citrus fruits, berries, plums, tropical fruits, and stone fruits [6].

Several factors influence fruit production worldwide. Climatic conditions such as temperature, rainfall, solar radiation, humidity, and seasonal variations are among the most critical determinants affecting fruit yield and quality. Climate change has emerged as a major challenge for fruit cultivation because extreme weather events, droughts, heat stress, and irregular precipitation patterns negatively impact fruit productivity and postharvest stability [4]. Soil fertility, irrigation management, pest and disease incidence, cultivar selection, and agricultural practices also strongly influence fruit production efficiency and quality characteristics [4,30].

In addition to environmental factors, socioeconomic and technological aspects play important roles in fruit production systems. Access to modern agricultural technologies, storage facilities, transportation infrastructure, and market availability directly affects the profitability and sustainability of fruit industries [4,31]. Postharvest losses remain a major global problem, particularly in developing countries where inadequate storage and processing infrastructure can result in significant economic losses and food waste [31].

Fruit processing has therefore become increasingly important for extending shelf life, reducing postharvest losses, improving product stability, and generating value-added products. Processing technologies allow fruits to be transformed into juices, jams, concentrates, dried fruits, fermented beverages, and wines, thereby enhancing market diversification and economic opportunities for producers. Fruit wine production represents one of the most promising approaches for utilizing surplus fruits and underexploited varieties while simultaneously promoting sustainable agro-industrial development and circular economy practices. Furthermore, fruit processing contributes to the preservation of nutritional and bioactive compounds, including vitamins, phenolics, flavonoids, and antioxidants, which are associated with important health benefits [32].

### 3.2. Technological Approaches Applied During the Production of Fruit Wines

**Figure 2:**
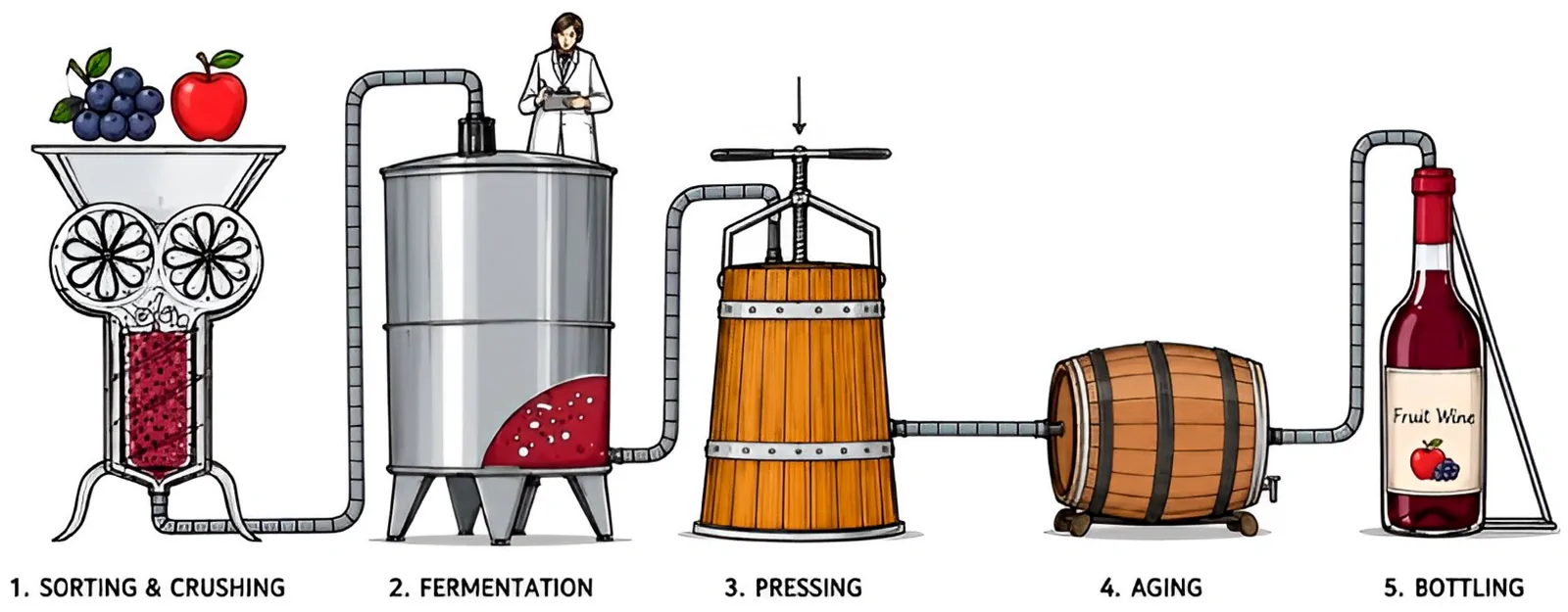
Fruit wine production process.

The production of fruit wines involves several technological stages designed to optimize fermentation performance, preserve sensory quality, and improve the stability of the final product. Although fruit wine production shares many similarities with grape winemaking, important technological modifications are often necessary due to differences in sugar content, acidity, tannin levels, and nutrient composition among fruits (Figure 2) [11,30].

The process generally begins with fruit selection, sorting, washing, peeling (when necessary), crushing, and juice extraction. Pre-treatment operations are essential to remove contaminants and improve juice quality. Because many fruits contain lower sugar concentrations than grapes, chaptalization (sugar addition) is frequently applied to achieve adequate alcohol levels during fermentation. Similarly, acidification or deacidification may be required to adjust pH and optimize yeast activity [30]. Yeasts play a central role in fruit wine fermentation. The yeast species most commonly used is *Saccharomyces cerevisiae* because of its high ethanol tolerance, efficient sugar metabolism, and favorable aroma production. To obtain a well-balanced acidity and alcohol level in cider, simultaneous inoculation with *S. cerevisiae* and *Schizosaccharomyces pombe* ensures controlled alcoholic fermentation, resulting in a final product with optimal flavor. However, non-Saccharomyces yeasts such as *Torulaspora delbrueckii*, *Metschnikowia pulcherrima*, *Pichia kluyveri*, and *Hanseniaspora uvarum* have gained increasing attention due to their ability to enhance flavor complexity, glycerol production, and volatile aroma profiles [33,34]. Mixed fermentations involving both Saccharomyces and non-Saccharomyces yeasts are currently being explored to improve the sensory diversity and functional characteristics of fruit wines [33]. Different fermentation types can be applied during fruit wine production. Alcoholic fermentation is the primary process in which sugars are converted into ethanol and carbon dioxide under anaerobic conditions. In some cases, malolactic fermentation performed by lactic acid bacteria is used to reduce acidity and improve microbiological stability [11,30]. Depending on the desired product characteristics, fermentations may be conducted as spontaneous fermentations using native microbiota or as controlled fermentations using selected starter cultures. Controlled fermentations are generally preferred at the industrial scale because they provide greater reproducibility, fermentation efficiency, and product consistency [11].

Recent technological advances in fruit wine production include the application of enzymatic treatments, ultrasound-assisted extraction, pulsed electric fields, high-pressure processing, immobilized yeast systems, and temperature-controlled fermentations. Enzymes such as pectinases, cellulases, and hemicellulases are widely used to improve juice extraction, clarification, color release, and phenolic compound recovery. Emerging technologies are also being investigated to preserve bioactive compounds, improve antioxidant activity, and reduce energy consumption during processing [35]. Additionally, fermentation parameters such as temperature, oxygen availability, yeast nutrition, fermentation duration, and maturation conditions strongly influence the chemical composition and sensory properties of fruit wines. Careful optimization of these parameters is essential to obtain products with desirable aroma, taste, color, stability, and consumer acceptance.

### 3.3. Types of Fruit Wines and the Most Common Fruit Wines Today

Fruit wines can be classified according to the fruit source, alcohol content, sweetness level, production method, and fermentation characteristics. Depending on the raw material used, fruit wines may be produced from temperate fruits, berries, citrus fruits, tropical fruits, or mixed fruit combinations. They may also be categorized as still wines, sparkling wines, dessert wines, fortified wines, or low-alcohol fermented beverages.

Among the most commercially important fruit wines worldwide, apple wine and cider represent the largest production segment due to the extensive global cultivation of apples and strong consumer acceptance. As a fermented beverage, cider contains more nutrients than distilled apple liquors. Additionally, the long tradition of cider production in Europe has positioned this beverage as an important product widely accepted among consumers [36]. It is interesting to note that the term “cider” is used in England for fermented apple juice, while on the other side of the Atlantic Ocean, in the USA, it is called “hard cider.” European countries such as France, Italy, and Spain use the same name (with slight modifications according to the language), while in German-speaking countries the term “applewein” is used. According to its alcohol content, cider can be classified as soft (1–5%) or hard (6–7%) [37]. Berry wines produced from blueberry, strawberry, raspberry, blackberry, cranberry, and elderberry are also highly popular because of their intense color, pleasant aroma, and high antioxidant content [38].

In Asian countries, plum wine, lychee wine, and rice–fruit blended wines are widely consumed and represent important regional products. Tropical fruit wines produced from pineapple, mango, banana, guava, jackfruit, and passion fruit have become increasingly popular in Africa, Latin America, and Southeast Asia due to the abundance of these fruits and their appealing sensory characteristics. Pomegranate wine has also emerged as a high-value functional beverage because of its elevated polyphenol and anthocyanin content [21,22,23,24,39]. Citrus fruits such as orange and mandarin have also shown good potential for processing into fruit wine. The production of wine from these fruits is particularly characteristic of Turkey, one of the leading producers of citrus fruits [18,40].

In South America, particularly in Chile and Argentina, fruit wine production has experienced gradual expansion as a complementary sector to the traditional grape wine industry. Both countries possess highly favorable climatic conditions for fruit cultivation and are among the leading producers of berries, apples, cherries, peaches, plums, and other temperate fruits. Consequently, several fruit-based fermented beverages have emerged as artisanal and industrial products with growing local and international market potential. In Chile, fruit wines are commonly produced from blueberries, raspberries, blackberries, strawberries, apples, and cherries, especially in the southern regions where berry cultivation is highly developed. Blueberry and maqui berry wines have attracted special interest because of their high anthocyanin and antioxidant contents, which are associated with potential functional and health-promoting properties. Additionally, Chilean producers have explored the development of sparkling fruit wines and mixed fruit fermentations aimed at diversifying the beverage market and adding value to surplus fruit production. Argentina has also developed an emerging fruit wine sector associated with regional fruit industries. In Patagonia and other temperate production areas, apple and pear ciders represent important traditional fermented beverages. In northern Argentina, tropical and subtropical fruits such as passion fruit and citrus fruits have also been evaluated for fermented beverage production due to their distinctive aroma profiles and high sugar contents [41].

The continental climate in Serbia provides favorable conditions for fruit growing, while agrotechnical practices and cultivar selection ensure high yields of high-quality crops. Berry, drupe, and pome fruits are produced in significant quantities. Among the berries, it is important to highlight blackberry and raspberry, whose production has positioned Serbia as a leading European and global producer of these high-quality fruits. Blackberry wine is the most popular fruit wine in Serbia among consumers due to its acceptable sensory properties and beneficial health effects [15]. Plum, sweet cherry, and sour cherry are the most important drupe fruits produced in Serbia. Sweet cherries are mostly consumed fresh, while sour cherries are suitable for wine production, resulting in a commercial product with an exceptionally pleasant aroma. Conversely, plums are used mostly for brandy production. Apples, apricots, and peaches are primarily processed into juice, jam, and brandy, while commercial fruit wines are rarely available. The production of high-quality fruit provides favorable conditions for the development of value-added products in Serbia, such as fruit wines [19].

In recent years, consumer interest in functional beverages, natural products, and artisanal fermented drinks has promoted the expansion of niche fruit wines with enhanced nutritional and bioactive properties. Wines produced from aronia berries, acerola, dragon fruit, kiwi, and indigenous fruits are increasingly investigated for their antioxidant activity and potential health benefits. Furthermore, low-alcohol and probiotic fruit wines are becoming emerging trends within the beverage industry due to growing demand for healthier alternatives and innovative fermented products [21,22,23,24,35]. To comply with zero-waste principles, fruit wine pomace can be upcycled into various new products similarly to grape wine [42], thus making wine production more sustainable. Namely, a low alcoholic (4% to 8% *v*/*v*) fizzy drink can be prepared by adding water to grape pomace, followed by pressing and fermentation, which is stopped before completion to form bubbles. This drink has been known since the time of the ancient Romans and today is called by the French word “piquette” [43,44]. Similarly to piquette, pomace obtained after apple pressing during cider production can be used to produce the drink named “ciderkin” [44]. The diversification of fruit wine production not only contributes to product innovation and market expansion, but also provides an effective strategy for utilizing surplus fruits, reducing agricultural waste, and promoting sustainable agro-industrial development. Consequently, fruit wines continue to represent an important and expanding sector within the global fermented beverage market [35].

## 4. Composition and Nutritive Significance of Fruit Wines and Pomace

### 4.1. Physico-Chemical Parameters of Quality

To ensure a high-quality, stable, and tasty fruit wine, winemakers measure specific physical and chemical properties. The most critical parameters include alcohol content, pH, total titratable acidity, sulfur dioxide, and total soluble solids (which measure the amount of dissolved sugar and other solids in a liquid), expressed as Brix degrees (°Bx).

#### 4.1.1. Total Soluble Solids

Brix degrees are widely used to assess fruit ripeness, evaluate crop quality, and guide winemaking. Compared to grapes, the sugar content of other fruits is much lower. To produce wines with an ethanol content of 10 to 12 vol%, it is important to increase the sugar level to 18 to 21 °Brix in the fruit must before fermentation begins. The process of adding sugar to the fruit must before the start of fermentation is called chaptalization, which ensures the production of sufficient alcohol to help prevent wine spoilage [45]. In addition to chaptalization, fermentation time significantly affects the alcohol content of fruit wines. Blueberry wine vinifications with total soluble solids in the range of 21.25 to 25% showed the highest alcohol content after fermentation lasting 14.5–16 days [17]. Apricot wine was produced by adjusting the juice with sucrose to 22 °Brix. Alcohol content was monitored over different days, and the highest value (11.4% vol) was reached after 8 days of fermentation [46]. Different yeast cultures applied during the fermentation of dragon fruit juice, which was previously adjusted to 20 °Brix by adding sucrose, produced wines with alcohol contents in the range of 8.25–8.93 %vol [21].

#### 4.1.2. Alcohol Content

Long-term fruit wine stability and protection against spoilage are associated with its alcohol content. As a solvent produced naturally during fruit fermentation, alcohol plays a key role in extracting phenolic compounds from the skins and seeds of the fruit. Fruit wine contains many different alcohols, but the most predominant among them is ethanol (Table 1). Additionally, during the fermentation process, aromatic and higher alcohols are generated, as well as ethanol esters, which contribute to the balance of acidity and fruit flavors [47]. Apples naturally contain more acids than sugars, so producing apple wine with an alcohol content of up to 5 vol% requires chaptalization. Alcohol acts as a natural preservative and gives the wine “body”. It is important to highlight that the prevention of bacterial population development can be achieved in wine with a pH of 2.9 and an alcohol content of up to 16 vol% [48]. Studies from Serbia showed that the addition of sugar before the start of fermentation was responsible for the higher alcohol content of wine, which increased the extraction of phenolic compounds and the antioxidant properties of blueberry, black chokeberry, blackberry, raspberry, and apple wines [15,49]. Compared with the results from Serbia, another study reported a much wider range for ethanol content, 4.5–11.1 vol%, in raspberry wines. This can be explained by the fact that different cultivars, grown under different climatic and agronomic conditions, were used for production [25]. Different technological approaches applied during alcoholic and malolactic fermentation of black raspberry showed that ethanol content increased in wines produced using controlled malolactic fermentation [50]. In strawberry wine produced after the addition of pectin to the fruit must and subsequent enzymolysis before fermentation, an alcohol content of 5.99% was achieved [25]. A study from Slovakia revealed that fermentation of strawberry must, to which sucrose was added beforehand, resulted in an alcohol content of 16.7% in the obtained wine [51]. Drupe fruits with dark skin (plum, sweet cherry, and sour cherry), as well as those with bright skin (peach and apricot), had higher alcohol contents in wines produced with the addition of sugar before the start of fermentation. This indicates that it is important to measure the initial sugar content in fruit juice to obtain an adequate ethanol content through chaptalization [19]. Another study indicated that peach wine produced from juice and pulp had different alcohol contents (5.11–12.76 vol%), which was attributed to the addition of water during fermentation that significantly decreased the ethanol content compared to wine to which no water was added [20]. Before the start of fermentation of kiwi juice, the sugar content was adjusted by the addition of sucrose, while different yeasts were applied during fermentation. This approach resulted in different alcohol contents in kiwi wines [52]. Additionally, wines produced by the same technological approach from different kiwi cultivars showed a wide range of alcohol contents (6.0–14.0%) [53]. Pineapple wines produced after alcoholic and malolactic fermentation had different alcohol contents depending on whether they were produced from pulp alone or from pulp with the addition of residues [24]. Fermentation of longan juice with different yeast strains resulted in different alcohol contents in longan wines, due to the different properties of the yeasts used in production [23].

#### 4.1.3. pH Value

The pH of wines produced from grapes ranges from 2.8 to 4. The pH of fruit wines can vary and mostly depends on the fruit used for their production (Table 1). For microbial safety in fruit wine, the target pH range is 3.0–3.8. Wines with a high pH above 4.0 are fragile, more prone to bacterial spoilage, and are more susceptible to oxidation (turn brown). Conversely, wines with a lower pH have a crisp taste and better storage stability. In addition, a lower pH is important for preventing bacterial growth in wine [3]. Additionally, the pH value of blueberry wine depends on TSS content and fermentation time. The highest pH values were observed in a vinification process in which TSS was in the range from 22.81 to 28.11%, whereas fermentation lasted from 8.76 to 17.24 days [17]. The pH value of peach wine, in which pulp was diluted with water before the start of fermentation, showed higher pH values compared with wines produced without prior water addition [20]. The pH value of apricot wine decreased during fermentation and became constant at the end of the process, reaching 3.8, which is important for protecting wine from the development of undesirable microorganisms [46]. Compared with the pH value of kiwi juice, the fermentation process increased the pH value, while the pH values of wines produced from different kiwi cultivars also increased, with some wines reaching values similar to those of the juice [53,56]. Different yeast strains used during the fermentation of longan juice were responsible for different pH values of longan wine, which can be attributed to the specific characteristics of the yeasts [23].

#### 4.1.4. Total Titratable Acidity (TTA)

Where pH measures the strength of acids, TA measures the total quantity of titratable acids. Acidity prevents the wine from tasting flat or flabby, and is a major structural component of fruit wine. The presence of specific organic acids in fruit depends on the type, whereas malic acid is the most predominant in drupe and pome fruit, while in citrus fruits, citric acid predominates [52]. During the fermentation of fruit must, several organic acids are produced, such as lactic, acetic, propionic, and butyric acids. It is important to emphasize that wine maturation, organoleptic properties, and the stability of fruit wine depend on pH value and acid content [57]. Due to the varying acid content of different fruits, there is a corresponding variation in TTA content in fruit wines (Table 1), which significantly affects sensory properties. Additionally, the amount of free SO_2_ is significantly affected by wine acids, which is important for the microbial stability of wine. A study conducted in China on consumer preferences for fruit wines showed that blueberry wines from four different producers, with total titratable acidity in the range of 7.4–14.9 g/L, were perceived as having a balanced taste between sweetness and sourness [54]. The total titratable acidity of black raspberry wines produced under different fermentation conditions revealed that controlled malolactic fermentation was responsible for producing wine with lower values of this parameter, which is important for the sensory properties of the final product [55]. The total titratable acidity of strawberry wine from Poland was lower than that from Serbia, which can be attributed to different approaches to fruit breeding as well as to the processes applied during wine production [19,38]. The use of different apple cultivars for wine production in Poland resulted in varying values of total titratable acidity, ranging from 3.32 to 4.31 g/L [51]. The addition of water to peach must during fermentation significantly decreased the total titratable acidity compared with wines to which no water was added before the start of fermentation [20]. The content of total titratable acids during an 8-day fermentation period continuously increased up to 9 g/L, which is important for the stability of fruit wine and the subsequent maturation process [46]. Total titratable acids were higher in wine than in kiwi juice, which can be explained by the production and degradation of different organic acids through various biochemical pathways during vinification. The use of different kiwi cultivars affected the content of total titratable acids in the wines, which could be explained by the different properties of the cultivars used in vinification [53,56].

#### 4.1.5. Free SO_2_ Content

In the beverage and food industry, one of the most common antimicrobial and antioxidant agents is SO_2_. Wine spoilage is prevented by free SO_2_, while another portion is bound to wine biomolecules. The presence of SO_2_ prevents oxidation and the growth of unwanted bacteria and yeasts, keeping wine fresh and preventing it from turning into fruit vinegar over time [58]. In fruit must, K_2_S_2_O_5_ is added before the start of alcoholic fermentation. During the vinification process, free SO_2_ is maintained at levels that are important for controlled fermentation by *Saccharomyces cerevisiae*. Determining free SO_2_ content is important for both technological and health reasons (Table 1).

### 4.2. Biological Active Compounds-Phenolic Profile of Fruit Wine and Fruit Pomace

**Figure 3:**
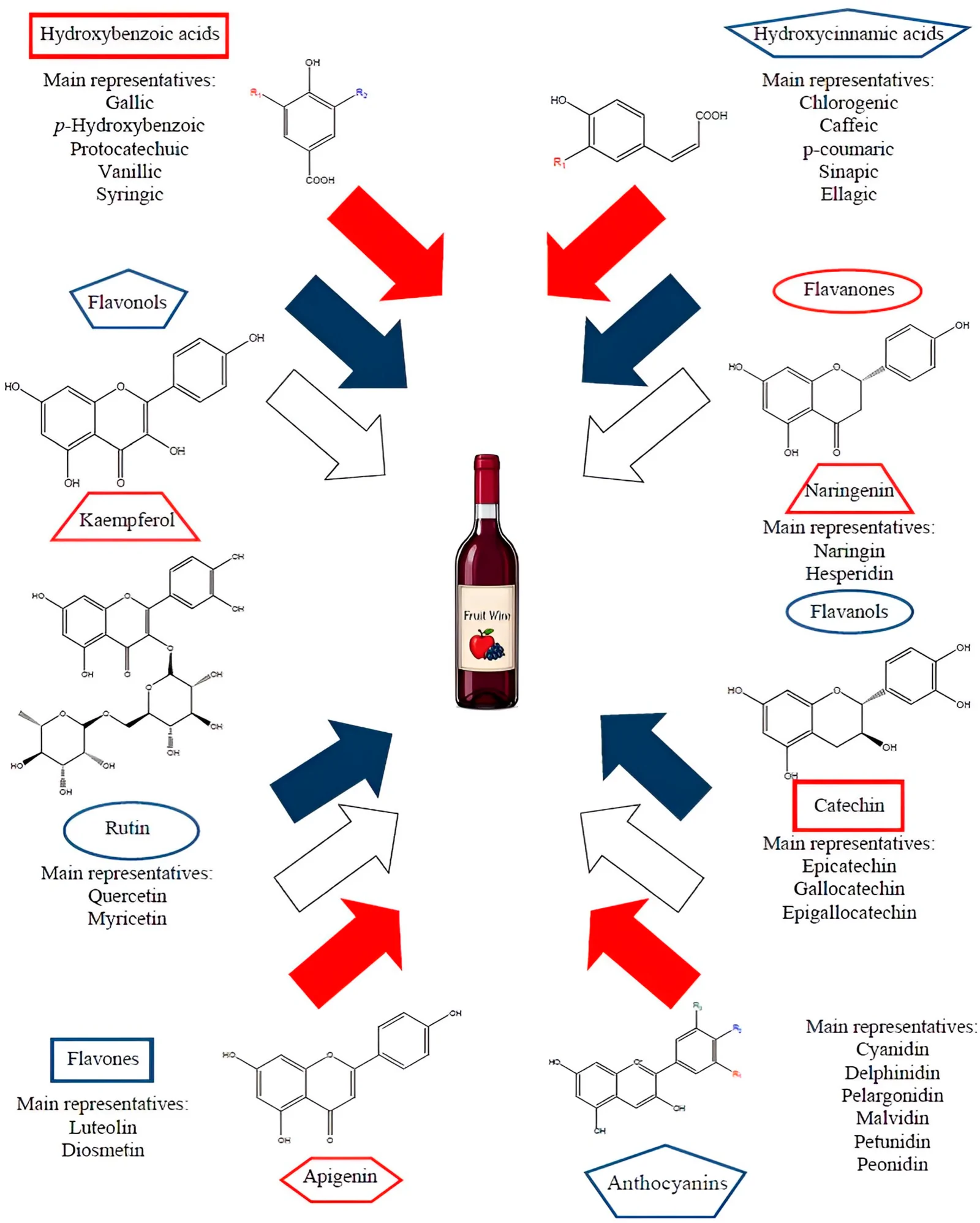
Phenolic compounds present in fruit wines.

Phenolic compounds are among the most important bioactive constituents of fruit wines, largely determining their antioxidant capacity, color, sensory properties, and overall biological value [38,55,59]. They comprise three major classes, namely phenolic acids, flavonoids, and anthocyanins (Figure 3), whose qualitative and quantitative composition is primarily governed by fruit species and genotype [19,38,55]. Their content significantly influences the biological activity of fruit wine and pomace (Table 2).

Studies from Serbia that investigated the antioxidant properties and phenolic profiles of fruit wines found higher TPC in blueberry, blackberry, black chokeberry, raspberry, and strawberry wines in which the alcohol content was increased by chaptalization. Higher TPC was strongly correlated with the FRAP and DPPH values of the aforementioned wines. This can be explained by the fact that higher alcohol content improves the extraction of phenolic compounds from the solid parts of the fruit (skin and seeds) during fermentation. The highest TPC was found in black chokeberry wines, followed by blueberry and blackberry wines [15,49]. Conversely, a study from the USA showed higher TPC in wines produced from blackberry than from blueberry, which contributed to the higher antioxidant activity determined by the ORAC method for blackberry wine. Such findings may result from the selection of fruit cultivars used for wine production, as well as from the technological approach [16]. These findings from the USA were consistent with a Polish study that reported lower TPC in blueberry wine than in blackberry, strawberry, and raspberry wines [38]. Two studies that investigated the antioxidant capacity and phenolic profile of apple wines showed that the TPC of the analyzed wines depended on the ethanol content and variety used for production. Higher ethanol content improved the extraction of phenolic compounds, while different cultivars, by their nature, have different phenolic contents. Additionally, all of these factors significantly affected the FRAP, ABTS, and DPPH values of apple wines, indicating an important contribution of phenolic compounds to their biological activity [15,51].

Fruit wines produced from drupes with dark skins (plum, sweet cherry, and sour cherry) showed higher TPC than those produced from fruits with brighter skins (peach and apricot). Additionally, in all wines produced from drupe fruits, increased alcohol content led to higher TPC values and greater antioxidant and antiradical activity [19]. Different TPC values in red araca wines resulted from the production process, in which varying pH values of the fruit must and the addition of potassium metabisulfite at pre- or post-fermentation stages affected phenolic content. In longan wines, different yeasts used for fermentation were responsible for varying TPC values. The different TPC values of red araca and longan wines did not correlate with antioxidant activity, which indicates the presence of biologically active compounds other than phenolics that contribute to the antioxidant properties of these two wines [23,69]. Additionally, in kiwi wines, the use of different cultivars resulted in differences in TPC values [56]. A study of fig wine showed higher TPC and TFC in the wine than in fig pulp. During six months of wine aging, the TPC values increased during the first two months and then decreased, while TFC decreased over the six months. The same pattern was observed for DPPH values of fig wine, which can be explained by the fact that phenolic compounds significantly affect biological activity [68].

Anthocyanins, the phenolic compounds responsible for red, purple, and blue coloration, were present in significant amounts in fruit wines produced from berries with dark skins. The TAC and TFC of blackberry wines depended on the geographic origin of the wine, indicating a significant influence of climatic conditions and breeding approaches on the anthocyanin and flavonoid content in the fruits used for wine production. Additionally, the content of biologically active compounds from these two groups significantly contributed to the antioxidant activity of blackberry wines determined by the ABTS and DPPH methods [63]. Different technological approaches applied during the processing of black chokeberry led to varying TAC values in the obtained products [61]. In a study of blueberry wines from Mexico, TAC was detected after an optimized production process [17]. Strawberry wines produced after mash fermentation had higher TAC and FRAP values than those produced from juice. This can be explained by the fact that anthocyanins are present in strawberry skins and are extracted during mash fermentation [64].

However, numerous agronomic and technological factors applied during the production process may substantially affect the extraction, transformation, and stability of these compounds during vinification, ultimately shaping the phenolic profile of the final wine [15,19,20,59].

#### 4.2.1. Phenolic Acids

Phenolic acids represent the predominant non-flavonoid polyphenols in fruit wines and mainly comprise hydroxybenzoic acids (e.g., gallic, p-hydroxybenzoic, protocatechuic and vanillic acids) and hydroxycinnamic acids (e.g., chlorogenic, caffeic, ferulic and p-coumaric acids). Owing to their wide distribution in fruits, they are considered one of the main contributors to the characteristic phenolic fingerprint of fruit wines [15].

##### Berry Fruit

Among all fruit wines, berry wines exhibited the greatest diversity of phenolic acids, although marked interspecies differences were observed. Hydroxycinnamic acids generally predominated over hydroxybenzoic acid derivatives, particularly in blueberry wines, where chlorogenic acid was the major phenolic acid. Similarly, chokeberry wines were characterized by high concentrations of chlorogenic, protocatechuic, p-coumaric, and caffeic acids, whereas catechin and ellagic acid occurred at comparatively lower levels. In contrast, hydroxybenzoic acid derivatives predominated in raspberry wines, with gallic acid identified as the major phenolic acid [15]. Blueberry wines exhibited a distinct phenolic acid profile dominated by caffeic acid and caffeic acid hexoside, which together accounted for nearly 75% of the total phenolic acid content, while elderberry wines were characterized by a more diverse hydroxycinnamic acid composition. Unlike bilberry and raspberry wines, blackberry and strawberry wines were dominated by *p*-coumaroylhexoside, representing approximately 85% and 47% of the total phenolic acid fraction, respectively [38]. Furthermore, gallic acid was identified as the predominant polyphenolic compound in blackberry wines [63], whereas strawberry wines generally contained the lowest total phenolic acid levels among the berry fruit wines investigated [15]. Fruit genotype and cultivation system further contributed to phenolic acid variability. Blue bilberry wines accumulated significantly higher concentrations of phenolic acids than white cultivars, while organic blackberry wines contained higher concentrations of caffeic and p-coumaric acid than conventionally produced wines [63,70]. Berry wine pomaces also represent a significant source of phenolic acids. Blueberry wine pomace was shown to contain ellagic acid, but in concentrations lower than those found in blueberry juice pomace. Ellagic acid was also found in raspberry wine pomace, and could be successfully recovered from this by-product [60,71].

##### Drupe Fruit

Compared with berry wines, drupe fruit wines generally showed a less diverse phenolic acid composition, with chlorogenic acid consistently representing one of the predominant hydroxycinnamic acids. Among the investigated species, sweet cherry wines had the richest phenolic acid profile and antioxidant potential, with chlorogenic, gallic, *p*-hydroxybenzoic, vanillic, and caffeic acids identified as the principal phenolic acids in both sweet and Chinese cherry wines [19,72]. In peach wines, chlorogenic acid was also one of the dominant phenolic acids, together with gallic, caffeic, and ferulic acids, indicating that hydroxycinnamic acids largely determine their phenolic profile [20]. Similarly, small white apricot wine was characterized by the highest chlorogenic acid content among the investigated apricot wines [46]. In contrast, plum wines contained comparatively higher proportions of caffeic, p-coumaric, and vanillic acids than the other stone fruit wines [19]. Besides fruit species, vinification strategy also influences phenolic acid extraction, as sugar addition, pit inclusion, and pulp fermentation generally enhance the recovery of individual phenolic acids during winemaking [15,19,20].

##### Pome Fruit Wines

Among the pome fruit wines, apple cider is predominantly characterized by hydroxycinnamic acids, particularly chlorogenic acid, whereas gentisic and 2,3-dihydroxybenzoic acids are the principal hydroxybenzoic acids. Although the phenolic acid profile of cider is less diverse than that of berry wines, malolactic fermentation increases the complexity of individual phenolic acids, highlighting the importance of secondary fermentation in shaping cider composition [59]. In addition, the abundance of chlorogenic acid in ciders and apple juice was shown to be 6.2–10.7% of the total phenolic content present in the analyzed samples. The contents of chlorogenic and caffeic acids showed a good correlation with the total phenols in ciders and apple juice [73,74]. The influence of the fermentation process on the content of specific phenolic acids in cider is also noteworthy. Compared with apple juice, the contents of chlorogenic, gallic, p-coumaric, caffeic, and ferulic acids decreased, while protocatechuic acid increased in cider [75].

##### Citrus Fruit

The initial phenolic composition of orange fruit affects the phenolic profile of juice and wine. Vinification procedures applied during production are also responsible for the phenolic content, which can change due to solubilization and extraction. Orange wines contain both hydroxybenzoic and hydroxycinnamic acids, with gallic acid predominant among the hydroxybenzoic acids and ferulic acid being the major hydroxycinnamic acid, followed by sinapic, chlorogenic, caffeic, and p-coumaric acids [18]. Another representative of the citrus fruit group, mandarin, can also be used for wine production. It is interesting to highlight mandarin wine as a source of phenolic acids. In wines produced from three different mandarin cultivars, the total amount of hydroxycinnamic acids was detected at higher levels than that of hydroxybenzoic acid derivatives. From the first group of phenolic acids, the most predominant compound in all three wines was vanillic acid, while from the second group it was ferulic acid [40].

##### Tropical Fruit

Compared with other fruit groups, tropical fruit wines exhibited highly species-specific phenolic acid profiles. Kiwi wines were mainly characterized by protocatechuic, gallic, and ellagic acids, with protocatechuic acid representing one of the predominant phenolic acids, while cultivar and fermentation strategy influenced their relative abundance [53,56]. Red araçá wine exhibited a distinct phenolic profile, in which gallic acid was identified as the principal phenolic acid, accompanied by catechin and tyrosol as the major phenolic compounds [69]. The phenolic profile of mango wine depends significantly on the fruit cultivar used during the vinification process. Wine produced from the Alphonso mango variety had the highest total phenolic content. In the same wine, among the hydroxybenzoic acid derivatives, vanillic acid, syringic acid, and protocatechuic acid were detected, while p-hydroxybenzoic acid was found at the highest level among the compounds in this group. Hydroxycinnamic acid derivatives were present in considerable amounts, with caffeic acid, ferulic acid, and sinapic acid being the most predominant derivatives in this group [76]. Custard apple is a fruit suitable for processing into a fermented beverage, especially when ripe, to prevent postharvest losses. Analysis of the phenolic profile of custard apple wine revealed the presence of hydroxybenzoic acids (gentisic acid, protocatechuic acid, and gallic acid), while among the hydroxycinnamic acids, caffeic acid and p-coumaric acid were present [22]. Among the phenolic compounds in jackfruit wine, protocatechuic acid and gallic acid were detected [77].

#### 4.2.2. Flavonoids

Flavonoids represent one of the largest groups of phenolic compounds in fruit wines and mainly include flavan-3-ols (e.g., catechin, epicatechin, and procyanidins), flavonols (e.g., quercetin, kaempferol, myricetin and isorhamnetin derivatives), and in citrus fruits, flavanones such as hesperidin and narirutin. Due to their pronounced structural diversity, flavonoids substantially contribute to the antioxidant potential and sensory characteristics of fruit wines, while also serving as useful markers for distinguishing wines produced from different fruit species [5,18,19,38].

##### Berry Fruit Wines

Similar to phenolic acids, berry fruit wines represented the richest source of flavonoids, although their composition differed markedly among individual species. Blueberry wines exhibited the greatest flavonoid diversity, being particularly rich in quercetin, myricetin, isorhamnetin, catechin, epicatechin, and procyanidin derivatives [70]. Elderberry wines were characterized by abundant quercetin glycosides, whereas blackberry and strawberry wines accumulated comparatively higher concentrations of catechin, epicatechin, rutin, quercetin, and kaempferol [19,38]. Blueberry wines also represented an important source of catechin and epicatechin, while chokeberry wines generally exhibited a less diverse flavonoid composition than the other berry wines. In addition, blue bilberry wines contained significantly higher concentrations of flavonoids than white cultivars [15,70].

##### Drupe Fruit Wines

Drupe fruit wines generally exhibited lower flavonoid diversity than berry wines but remained valuable sources of flavan-3-ols and flavonols. Sweet cherry wines possessed the richest flavonoid profile, characterized by elevated concentrations of catechin, epicatechin, quercetin, kaempferol, and naringenin, whereas plum wines contained comparatively higher levels of rutin and quercetin derivatives [19]. Additionally, sour cherries are a rich source of different flavonoids that pass into the fruit wine after the vinification process [15,78]. In contrast, peach and apricot wines exhibited simpler flavonoid compositions, mainly dominated by catechin, rutin, and quercetin. Among the investigated technological approaches, fermentation with fruit pulp and pit inclusion generally promoted higher flavonoid extraction than juice fermentation alone [19,20,46].

##### Pome Fruit Wines

Apple cider is predominantly characterized by flavan-3-ols, particularly catechin, epicatechin, and procyanidins, together with quercetin and phloretin derivatives [59]. The fermentation process influenced the content of flavonoids such as catechin, (−)-epicatechin, and rutin, whose levels was lower in cider compared to apple juice [75]. Apple pomace from the cider industry had a composition similar to that of apple cider, containing, among other compounds, phloridzin, procyanidin B2, rutin, isoquercitrin, protocatechuic acid, and hyperin [62]. Japanese quince pomace was shown to be rich in epicatechin, procyanidin B2, and procyanidin C1 [62].

##### Citrus Fruit Wines

Citrus wines clearly differed from all other fruit wines due to the predominance of flavanones, particularly hesperidin and narirutin, accompanied by lower amounts of neohesperidin, didymin, and naringin [18,79]. These compounds are considered characteristic markers of citrus wines and largely contribute to their distinctive biological and sensory properties, including their yellow-orange color. Conversely, the extraction dynamics observed during 15 days of alcoholic fermentation of orange juice revealed the highest flavonoid content on day 11. During alcoholic fermentation, less-soluble flavonoids are increasingly extracted. Additionally, other technological factors, such as industrial squeezing and extraction pressure, could also contribute to this process [78,80]. Flavanones were the most predominant group of phenolic compounds detected in mandarin wines produced from three different cultivars. Among the specific compounds from this group, hesperidin was the most predominant in all wines, followed by narirutin as the second most abundant [40].

##### Tropical Fruit Wines

Tropical fruit wines exhibited pronounced species-specific flavonoid profiles. Kiwi wines were mainly characterized by catechin, epicatechin, quercetin, and quercitrin derivatives, while significant differences were observed among cultivars and fermentation methods [53,56].

#### 4.2.3. Anthocyanins

Anthocyanins are the principal pigments responsible for the characteristic red, purple, and blue colors of fruit wines. Their qualitative and quantitative composition is largely determined by fruit pigmentation and genotype, although fruit maturity and vinification conditions markedly influence pigment stability throughout fermentation and storage [38,69,70,72,79,81].

##### Berry Fruit Wines

Berry fruit wines exhibited the highest anthocyanin diversity among all of the investigated fruit wines. Blackberry wines were dominated by cyanidin-3-*O*-glucoside, whereas raspberry wines contained predominantly cyanidin-sophoroside and cyanidin-3-(2G-glucosylrutinoside). In contrast, strawberry wines were characterized by pelargonidin-3-*O*-glucoside as the principal pigment, confirming clear species-specific differences in anthocyanin composition [38]. Bilberry wines displayed the greatest pigment diversity, containing numerous cyanidin, delphinidin, malvidin, petunidin, and peonidin derivatives, while blue bilberry wines accumulated substantially higher anthocyanin concentrations than white cultivars [38,70]. Blueberry wine pomace was shown to contain various anthocyanins, including delphinidin-3-O-glucoside, delphinidin-3-O-arabinoside, petunidin-3-O-glucoside, cyanidin-3-O-arabinoside, cyanidin-3-O-glucoside, malvidin-3-O-glucoside, and malvidin-3-O-arabinoside [60,82]. Cyanidin-3-galactoside was the most abundant anthocyanidin determined in aronia wine pomace [62]. Among the tropical fruit wines, mulberry exhibited one of the richest anthocyanin profiles, containing numerous cyanidin, delphinidin, pelargonidin, peonidin, malvidin, and petunidin derivatives. Anthocyanin composition was strongly influenced by fruit maturity, with cyanidin-3-O-glucoside predominating in less mature fruits and pelargonidin-3-O-glucoside becoming more abundant at advanced ripening stages [81].

##### Drupe Fruit Wines

Compared with berry wines, drupe fruit wines generally contained lower anthocyanin concentrations. Cherry wines represented the richest source of total anthocyanins, which predominated and largely contributed to wine color and antioxidant properties [72]. Conversely, plum, peach, and apricot wines contained considerably lower pigment levels and were better distinguished by their phenolic acid and flavonoid composition than by anthocyanin diversity [19,20,46].

##### Pome Fruit Wines

Anthocyanins contributed only marginally to the phenolic profile of apple cider because of the naturally low pigment content of apples. Consequently, color development in cider is mainly associated with oxidative reactions occurring during fermentation and maturation rather than with native anthocyanin concentration [59].

##### Citrus Fruit Wines

In blood orange wines, fermentation promoted the formation of hydroxyphenyl-pyranoanthocyanins, accompanied by a decrease in native anthocyanins [79]. A study of blood orange wine revealed a significant anthocyanin content, with anthocyanins being the most abundant phenolic compounds. Among the specific anthocyanins quantified were glucosides of delphinidin, cyanidin, and peonidin. High anthocyanin content was also found in juice and wine obtained from blood orange. Alcoholic fermentation decreased the anthocyanin concentrations due to polymerization reactions between anthocyanins and other phenolic compounds, resulting in the formation of condensed structures [79].

##### Tropical Fruit Wines

Red araçá wines contain several anthocyanins, including cyanidin-3-glucoside, petunidin-3-glucoside, peonidin-3-glucoside, and malvidin-3-glucoside, with cyanidin-3-glucoside predominating in all samples. Moreover, lowering the fermentation pH to 2.8 and adding 100 mg K_2_S_2_O_5_ before fermentation significantly increased cyanidin-3-glucoside retention and lead to better preservation of the wine’s reddish color [69].

## 5. Biological Activity of Fruit Wines and Fruit Pomace

### 5.1. Antioxidant and Antiradical Properties

Fruit wine can be considered an aqueous ethanol solution that is rich in biologically active compounds released during the winemaking process. After wine consumption, these compounds become bioavailable and are absorbed in the gastrointestinal tract. It is important to point out that the aforementioned compounds are responsible for the antiradical and antioxidant properties of fruit wines, including their ability to scavenge free radicals (Table 3). The highest antioxidant activity measured by the FRAP method was determined for blackberry wine, while elderberry and blueberry wines showed lower activities. Antiradical activity estimated by the ABTS and DPPH methods showed high values for blackberry and blueberry wines. Two other wines produced from berries with red skin, raspberry and strawberry, showed considerable antioxidant activity [38]. Blackberry wine showed higher antioxidant activity determined by the ORAC method than blueberry wine, while no significant effect was observed for the mixture of these two wines [16]. The antioxidant and antiradical properties of fruit wines depend strongly on the fruit used for their production. Wines made from berry fruits with dark blue or black skin, such as black chokeberries, blackberries, and blueberries, show significantly higher antioxidant activity measured by the FRAP method than apple and raspberry wines. The antioxidant properties of the aforementioned fruit wines depend on the vinification process applied during production. Higher alcohol content can enhance the extraction of phenolic compounds from the skins and seeds of the fruit, while the use of enzymatic preparations cleaves glycosidic bonds and releases the aglycones of phenolic compounds. High antiradical activity of black chokeberry and blueberry wines has been demonstrated using the DPPH method. This biological activity originates from all compounds that exhibit antiradical activity [15,83].

Biologically active compounds responsible for the dark skin of drupe fruits such as plum, sour cherry, and sweet cherry contribute to the antioxidant and antiradical properties of wines made from these fruits. Higher anti-DPPH activity and FRAP values were reported in the aforementioned wines than in wines produced from apricot and peach [15,19]. Different fruit varieties used in vinification procedures can significantly influence the antioxidant properties of fruit wines. This was noted for peach and apple wines and their DPPH and ABTS values [20,51]. Antioxidant activities of fruit wines from Turkey showed the lowest activity for apple and apricot wines, while red berry wines (raspberry and strawberry) had moderate activity. The highest antioxidant activity was reported for cherry and blackberry wines [84]. Anti-DPPH activity depends on the maturation process; in fig wine, it initially increased and then decreased. This phenomenon can be explained by the fact that during wine maturation, various reactions lead to the formation and degradation of biologically active compounds [68].

Additionally, it is important to highlight certain autochthonous fruits that possess high antioxidant and antiradical activity, such as maqui berry, indigenous to the mountainous regions of Chile. Assessments of the antioxidant and antiradical activities of maqui juices and fermented products have reported relatively high values. Particular attention has been drawn to the alcoholic maceration and fermentation of this fruit because of its high levels of phenolic acids, proanthocyanidins, and anthocyanins, among other flavonoids. These high levels contribute to its antioxidant capacity compared with other berries [85,86], such as calafate (*Berberis mycrophylla*), which has a slightly lower concentration of phenols and antioxidant activity than maqui and other South American fruits [87]. Maqui contains more than 20 phenolic compounds, including anthocyanins (and anthocyanidins), as well as phenolic acids and flavonoids [86,88]. Furthermore, according to one study, maqui fruit contains more than 12 g gallic acid equivalents per kilogram of fresh fruit (g GAE kg^−1^ fresh fruit), significantly higher than the phenolic content of other cultivated species: twice as much as strawberries (*Fragaria* × *ananassa*), blueberries (*Vaccinium corymbosum*), and blackberries (*Rubus ulmifolius*) [89]. Moreover, another study reported [90] values from 1279 mg GAE/100 g FW in the maqui extract to 3592.7 mg GAE/100 g FW in samples concentrated by evaporation at 50, 70, and 80 °C when evaluating the total phenol content in mature maqui fruits, with values higher than those reported by other authors [91]. Its reputation as a species with extraordinary antioxidant capacity measured by ORAC, showing the ability to prevent oxidative damage to DNA, lipids, and other molecules, stems from its values exceeding 29,000 μmol TE 100 g^−1^ of fresh fruit (TE, Trolox equivalents). Only calafate (*Berberis microphylla*) surpasses these values with 25,662 μmol TE 100 g^−1^ of fresh fruit. Similarly, blueberries contain only 5481 μmol TE 100 g^−1^ of fresh fruit, slightly more than a quarter of the value of maqui berries. This has earned them the reputation of being the Chilean superfruit with the highest antioxidant and free-radical scavenging capacity of any berry in the world [85,86]. Additionally, it is important to highlight red araçá, an indigenous species from South America that could be used for wine production. In another part of the world, Southern China and Southeast Asia, longan fruits are processed into different products, one of which is wine. Observing values estimated by ABTS, FRAP, and DPPH methods for those wines, it is possible to highlight the significant influence of vinification procedures and the yeast used during fermentation on the antioxidant and antiradical properties of these wines [23,69].

### 5.2. Beneficial Health Effects

**Figure 4:**
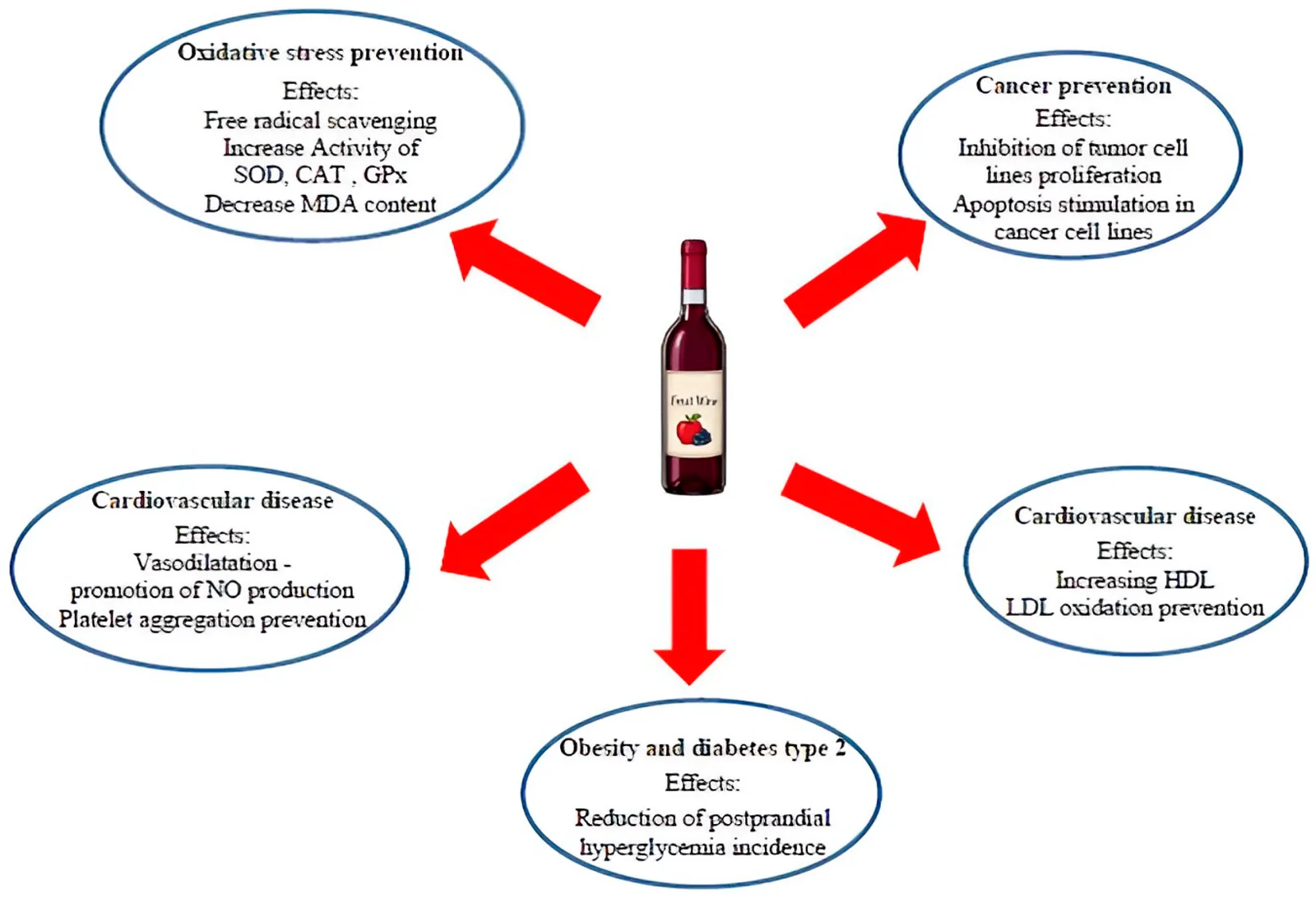
Health benefits of active compounds from fruit wines.

Unlike grape wine, whose health effects and profile of biologically active compounds have been studied in detail, fruit wines have received considerably less attention in this regard. Therefore, in addition to the potential beneficial effects of fruit wines (Figure 4), this section also discusses the reported health-related effects of fruit extracts and the fruits themselves that are used as raw materials for wine production. However, given the established health risks associated with excessive alcohol consumption, the discussion of potential benefits should not be interpreted as a recommendation to consume fruit wines or other alcoholic beverages.

#### 5.2.1. Absorption of Phenolic Compounds

The primary site of exposure to and absorption of phenolic compounds is the digestive system, and one of the main challenges is that their stability and bioavailability can be affected during digestion. The absorption and bioavailability of phenolic compounds may be influenced by the presence of ethanol, which is why wine, whether made from grapes or other fruits, has been investigated as a dietary source of these compounds. This is supported by findings that wines may provide greater bioavailability of phenolic compounds than isolated phenolic extracts or dealcoholized wines [92].

After absorption, phenolic compounds and their metabolites are transported through the circulation, partly bound to plasma proteins such as serum albumin, and are thus distributed to tissues, where they can undergo further metabolism. Among these compounds, it is important to highlight phenolic acids, which are subject to extensive phase II metabolism, including conjugation and O-methylation, primarily in the intestinal wall and liver, after which their metabolites can re-enter the circulation. Their metabolism is important because phenolic acids represent a substantial proportion of absorbed phenolic compounds (30–60%) and because some phenolic acids and their metabolites may contribute to radical-scavenging and other biological activities [93].

Glycosylated forms of anthocyanins can be detected in the bloodstream and target tissues within a few minutes of food intake. Anthocyanins from blueberries were absorbed at levels ranging from 19% to 37%, with delphinidin glycoside showing the highest absorption. It is important to note that consuming higher amounts of anthocyanins from blackberries does not necessarily increase their absorption in the body, which may be attributed to limitations in intestinal uptake, metabolism, and elimination rather than solely to a protective mechanism against high concentrations of phenolic compounds [94]. In contrast to blackberries, consumption of a strawberry-rich beverage resulted in various pelargonidin glycosides being the most abundant anthocyanins in human plasma [95].

Looking at the structures of flavonoids, only aglycones and some glycoside forms can be absorbed in the small intestine. Flavonoids are most often present in food as glycosides, or are polymerized or esterified with other biomolecules, which reduces their absorption [96]. Phenolic compounds act in the gastrointestinal system as free radical scavengers, thereby reducing the likelihood of lipid peroxidation. Flavonoids bound to sugars such as rhamnose must first be hydrolyzed by rhamnosidases found in the colonic microflora. Rhamnosides and other bound forms of flavonoids are generally less efficiently absorbed, despite the action of the appropriate enzymes in the colon, because absorption in this part of the digestive tract is lower than in the small intestine. Even so, compounds formed by the bacterial degradation of phenolic compounds show a positive effect in the colon [97,98].

Absorption of quercetin in the human body occurs in the digestive system, where various conjugated forms of this flavonoid, such as glucuronides and sulfates, can be detected in bile and urine. It is also important to point out that the structures of the aglycone and glycoside molecules lead to significant differences in their absorption [99]. The effect of alcohol on the absorption of catechins is also of interest. It was shown that the catechin content in plasma one hour after intake of 120 mL of red wine was 91 ± 14 nmol/L, whereas intake of the same amount of dealcoholized wine resulted in a plasma catechin content of 81 ± 11 nmol/L [100]. Many studies highlight large interindividual differences in flavonoid absorption, primarily due to polymorphisms of digestive system enzymes and other transport proteins [101]. These findings indicate that the positive health effects of different flavonoids depend on their kinetics of absorption and elimination, as well as on the form in which they are present in the human body.

#### 5.2.2. Oxidative Stress Prevention

Oxidative stress is associated with the development of many chronic non-communicable diseases. The mechanism involves the disruption of redox homeostasis, leading to increased levels of free radicals that trigger pathological conditions in the body. The antioxidant system consists of enzymatic antioxidants (SOD, CAT, GPx) present in cells, which maintain homeostasis in our body through their activity. Antioxidants such as phenolic compounds and vitamins are ingested daily with the food we consume; they can scavenge reactive species and may also modulate the activity of antioxidant enzymes through different signaling pathways [94].

Fruit wines have been shown to reduce the negative impact of experimentally induced oxidative stress on cells. In a model system using synaptosomes obtained from rat brains, oxidative stress was induced with hydrogen peroxide. After oxidative stress was induced and the synaptosomes were treated with fruit wines, the activities of SOD, CAT, and GPx increased. Among wines produced from berry fruits, the activities of these enzymes increased the most in wines made from blueberries, blackberries, and strawberries. Wines made from drupe fruits, such as sweet cherries and plums, also showed a strong increase in enzyme activity. Under experimentally induced oxidative stress, fruit wines reduced the level of MDA (malondialdehyde), helping prevent damage to lipids that are part of the cell membrane. The strongest reduction in MDA levels was observed with plum and chokeberry wines [102,103]. The previously described properties of wine originate from phenolic compounds that can influence the activity of antioxidant protection enzymes and reduce oxidative stress. Oxidative stress underlies the development of some neurodegenerative diseases, so the presence of phenolic compounds can influence prevention. After absorption in the digestive system, phenolic compounds cross the blood–brain barrier, and their presence has been confirmed in the brain tissue of experimental animals [104]. The passage of polyphenols through the blood–brain barrier depends on lipophilicity: less polar metabolites (formed by O-methylation) pass more easily than polar ones (sulfates and glucuronides). In a cell model, catechin, epicatechin, naringenin, and gallic and ellagic acids have been detected in brain tissue, where they exhibit neuroprotective properties [105,106]. Another model system using fibroblasts (V79-4) obtained from Chinese hamster lungs demonstrated the ability of fruit wines to reduce the negative impact of oxidative stress. Experimentally induced oxidative stress by hydrogen peroxide in V79-4 cells decreased after treatment with wine and sour cherry juice. It is important to note that compared with juice and fresh fruit, wine produced a greater increase in the activity of the antioxidant protection enzymes SOD and CAT. This is because during fermentation, the content of biologically active compounds in wine increases compared with other cherry products [8].

#### 5.2.3. Cardiovascular Disease Prevention

Research on the cardioprotective properties of wine has mostly examined the effects of grape wine, primarily red, while studies using fruit wines are very rare. The vasodilatory effect of blackberry wine was significantly greater than that of white wines, while the effect of red wines was significantly higher than that of both white and blackberry wines. This activity of fruit wine is attributed to phenolic acid derivatives of hydroxycinnamic and hydroxybenzoic acids, whose vasodilatory properties have been experimentally confirmed [107,108].

Some phenolic compounds have proven to be highly effective in the potential prevention of atherosclerosis. One of the causes of this pathological condition is the loss of proper vascular endothelial function. The endothelium produces nitric oxide (NO), the most potent natural vasodilator. When endothelial function is impaired, wine consumption can improve its activity, promote vasodilation, and prevent platelet aggregation. In one study, berries and their products reduced platelet aggregation by as much as 11% compared to the control group. In this way, they significantly contribute to lowering blood pressure, increasing HDL cholesterol concentration, and influencing NO metabolism [109]. Phenolic compounds from wine can bind to the LDL fraction of cholesterol and thus prevent its oxidation, which is one of the causes of this pathological condition. Red wine has been shown to reduce LDL oxidation in plasma, with flavonoids, particularly quercetin, being significant contributors to this activity [110]. It is also important to note that one mechanism that may explain the preventive role of polyphenols in cardiovascular disorders is their ability to downregulate the expression of genes involved in regulating cell adhesion. This reduces the activity of circulating monocytes, which are responsible for the onset of inflammation when they are embedded in the vascular endothelium [111].

#### 5.2.4. Anti-Tumor Properties

Studies related to the antitumor properties of fruit wines are rare, while the potential of fruits and fruit extracts in the prevention of these diseases has been much more extensively studied. As rich sources of various phenolic compounds, berry fruit extracts have shown antiproliferative properties. Experiments on different tumor cell lines demonstrated that increasing the concentration of strawberry, blackberry, blueberry, raspberry, black raspberry, and cranberry extracts increased the inhibition of cell proliferation, with effects varying among cell lines. Strawberry and black raspberry extracts showed the strongest ability to stimulate apoptosis in the colon cancer cell line HT-29 [112]. The antiproliferative properties of black raspberry wine, juice, and extract have been demonstrated in colon and prostate cancer cell lines, where the suppression of proliferation was observed. The presence of black raspberry seeds, a significant source of biologically active compounds, increased the antiproliferative properties of the juice in cell lines, while similar effects were also observed for wine. In addition, the high ethanol content of 60% in the seed-rich extract improved extraction efficiency and enhanced the antiproliferative properties [113].

#### 5.2.5. Anti-Hyperglycemic Properties

Berries and their products may help reduce symptoms of metabolic syndrome, which underlies the development of obesity and type 2 diabetes [114]. The previously noted beneficial effect on the human body is attributed to various biologically active compounds present in grape and fruit wines. As a complex mixture, the synergistic action of these compounds is responsible for the positive effects on the human body. This was confirmed by a study showing that fruit wines can inhibit the enzyme alpha-glucosidase. This enzyme plays an important role in the final stage of carbohydrate digestion, so its inhibition reduces the incidence of postprandial hyperglycemia. Technological procedures applied during vinification produced fruit wines with a high content of phenolic compounds that exhibited significant inhibitory activity against alpha-glucosidase. Black chokeberry and blueberry wines showed the strongest inhibitory activity [15].

## 6. Future Perspectives

Despite the increasing number of studies on fruit wines, several aspects still require further investigation. A key priority is the standardization of analytical methods, as differences in extraction procedures, analytical techniques, units of measurement, and experimental conditions currently limit comparisons among various fruit wines and their pomaces. Future research should also focus on systematically optimizing fermentation conditions, including factors such as fruit characteristics, pH levels, the addition of sulfur dioxide, fermentation temperature and duration, enzymatic treatments, and yeast selection, with the aim to maximize the retention of bioactive compounds while ensuring desirable quality.

Additional studies should combine phenolic profiling with multivariate analysis to identify the specific compounds or groups of compounds that most accurately explain biological activity. There should also be greater emphasis on the biological significance of fruit-wine phenolics, especially regarding their bioaccessibility, bioavailability, and metabolic transformation after gastrointestinal digestion. Fruit wine pomace presents another promising area for research, as it retains significant amounts of bioactive compounds and serves as a valuable resource for further valorization. Finally, future studies should merge technological and chemical characterization with sensory evaluation, consumer acceptance, techno-economic analysis, and environmental assessment. This multidisciplinary approach will be essential for transforming the demonstrated potential of fruit wines and their pomaces into sustainable, economically viable, and commercially relevant products.

## 7. Conclusions

Fruit wine production is a multidisciplinary process that draws on knowledge and skills in food science, fermentation technology, biochemistry, sustainability, and agro-industrial innovation, with the aim of obtaining a high-quality end product. As fermentation-derived products, fruit wines and the pomace remaining after production are rich sources of biologically active compounds, supporting their consideration as potential functional foods. Fruit wine production represents an increasingly important sector within the global food and beverage industry because of its capacity to add value to fruit crops, reduce postharvest losses, and promote sustainable agro-industrial development. The growing availability of diverse fruits worldwide, combined with increasing consumer demand for innovative, functional, and artisanal beverages, has driven significant advances in fruit wine research and production technologies. Fruit wines not only provide an alternative use for surplus and underutilized fruits, but also contribute to the diversification of local economies and the promotion of circular economy strategies through the valorization of agricultural resources and by-products such as fruit pomace.

## Figures and Tables

**Table 1:** Physicochemical properties of some fruit wines.

Fruit Wine	Alcohol Content (Vol %)	Free Sulfur Dioxide Content (mg/L)		pH	Total Titratable Acidity (g/L)	References
Berry Fruit						
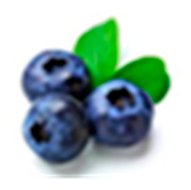 Blueberry (*Vaccinium myrtillus*)	7.88–11.31	10.24–16.66	n.r.	2.84–2.94	6.55–8.28	[15,17,25,38,49,54]
	12.86	n.r.	n.r.	n.r.	n.r.	
	7.63	n.r.	n.r.	n.r.	n.r.	
	2.5–12.05	n.r.	n.r.	2.91–3.01	n.r.	
	n.r.	n.r.	n.r.	n.r.	7.4–14.9	
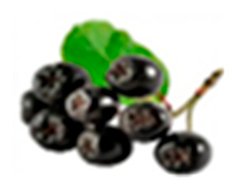 Black chokeberry (*Aronia melanocarpa*)	5.51–11.23	12.80–26.14		3.45–3.66	8.97–10.70	[15,49]
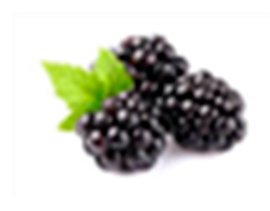 Blackberry (*Rubus fructicosus* L.)	7.63–10.43	10.24–18.59		2.81–2.94	6.42–9.32	[15,38,49]
	14.39	n.r.		n.r.	n.r.	
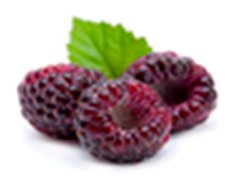 Black raspberry (*Rubus* spp.)	9.88–11.43	n.r.		3.19–3.28	8.73–10.77	[55]
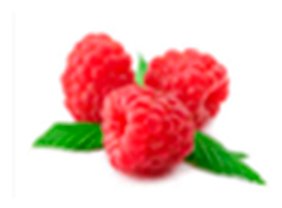 Raspberry (*Rubus idaeus*)	7.27–9.81	8.23–25.59		2.85–3.30	10.00–13.80	[15,25,38,49]
	8.43	n.r.		n.r.	n.r.	
	4.5–11.1	n.r.		2.9–3.05	3.94–17.65	
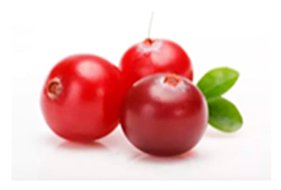 Cranberry (*Vaccinium* spp.)	7.22	n.r.		n.r.	n.r.	[38]
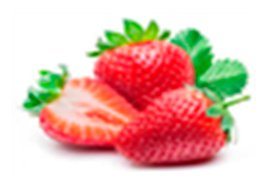 Strawberry (*Fragaria* × *ananassa*)	8.61–11.70	n.r.		3.24–3.40	8.91–9.55	[19,25,38,50]
	11.32	7.69–11.34		n.r.	n.r.	
	5.99	n.r.		2.8	3.27	
	16.7	n.r.		3.38	8.47	
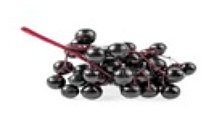 Elderberry (*Sambucus spp.*)	14.59	n.r.		n.r.	n.r.	[38,50]
	19.49	n.r.		3.98	4.75	
Pome fruit						
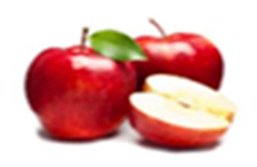 Apple (*Malus domestica* L.)	7.32–9.97	12.32–15.35		3.25–3.79	4.14–5.31	[15,49,50,51]
	10.99	n.r.		n.r.	3.47	
	n.r.	n.r.		n.r.	3.32–4.31	
Drupe fruit						
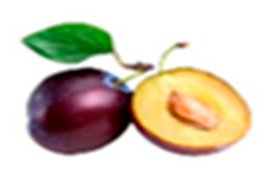 Plum (*Prunus domestica* L.)	7.17–9.17	9.41–13.03		3.31–3.67	9.46–10.78	[19]
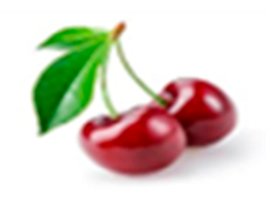 Sweet cherry (*Prunus avium* L.)	7.88–11.07	9.18–14.54		3.45–3.67	6.85–7.12	[19]
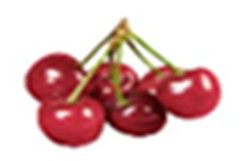 Sour cherry (*Prunus cerasus* L.)	6.79–11.31	9.45–20.48		3.20–3.80	6.90–8.90	[15,49]
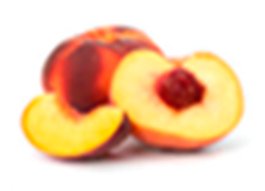 Peach (*Prunus persica* L.)	7.89–10.29	9.1–13.3		3.87–4.10	6.15–6.68	[19,20]
	5.11–12.76	15.51–32.35		3.30–3.36	4.93–5.12	
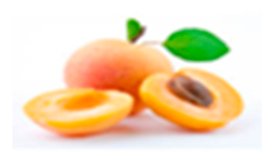 Apricot (*Prunus armeniaca* L.)	7.37–10.57	7.36–11.84		3.72–3.91	7.55–8.25	[19,46,50]
	11.4	n.r.		3.8	9.0	
	n.r.	n.r.		n.r.	9.9	
Citrus fruit						
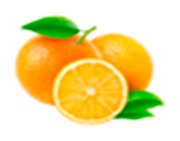 Orange (*Citrus* × *sinensis*)	12.6	8.2		3.6	6.3	[18]
Tropical fruit						
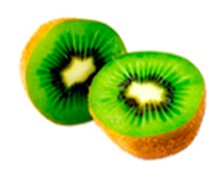 Kiwi (*Actinidia chinensis*)	6.0–14.0	n.r.		3.31–3.45	15.39–20.76	[53,56]
	5.30–5.50	n.r.		3.39–3.43	12.27–13.16	
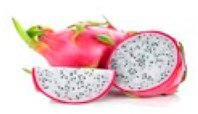 Dragon fruit (*Selenicereus undatus*)	8.25–8.93	n.r.		4.10–4.11	n.r.	[21]
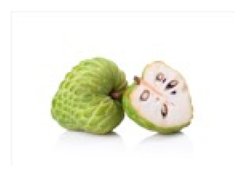 Custard apple (*Annona* spp.)	11.89	n.r.		4.23	n.r.	[22]
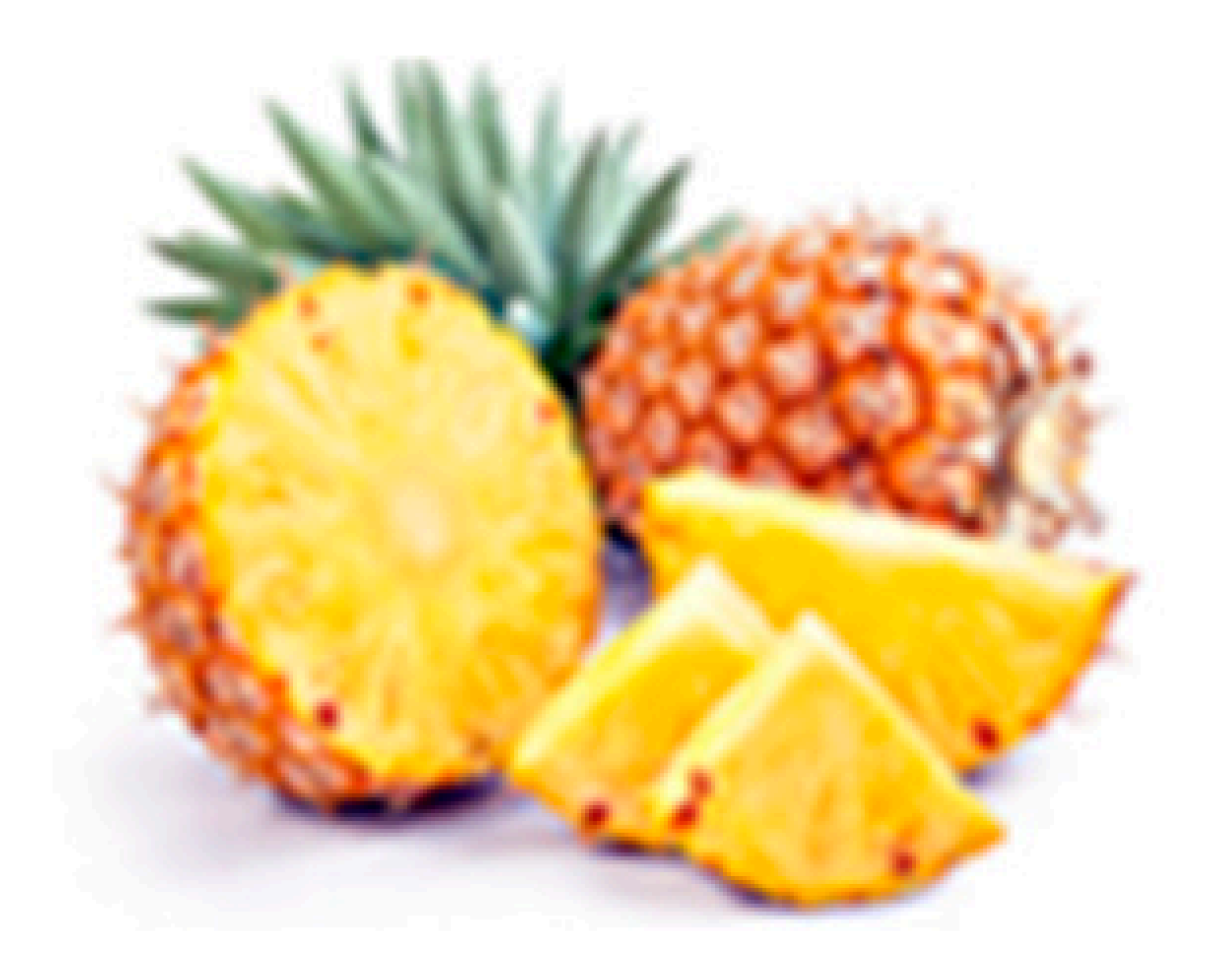 Pineapple (*Ananas comosus*)	7.5–10.84	n.r.		3.52–4.33	9.16–10.85	[24]
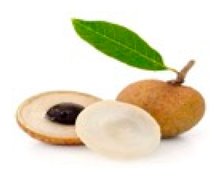 Longan (*Dimocarpus longan*)	7.93–11.20	n.r.		3.43–4.04	n.r.	[23]

Note: n.r.—not recorded.

**Table 2:** Content of phenolic compounds in some fruit wines and pomace.

Fruit Wine	TAC	TFC	TPC (mg GAE/L)	References
Berry Fruit				
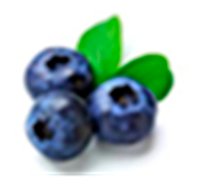 Blueberry (*Vaccinium myrtillus*)	n.r.	n.r.	2259–2459	[15,16,17,38]
	n.r.	n.r.	466.82	
	46.27 mg Cy3Gl/L	n.r.	360.27	
	76.8 mg/L	n.r.	2196.7 mg EAE/L	
Blueberry wine pomace	20.37 ± 2.59 mg C3GE/g	88.20 ± 7.63 mg RE/g	159.11 ± 13.31 mg GAE/g	[60]
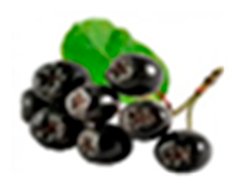 Black chokeberry (*Aronia melanocarpa*)	n.r.	n.r.	2351–2520	[15,61]
	1684–11,573 mg/100 g	n.r.	6310 mg GAE/100 g	
Black chokeberry wine pomace	n.r.	n.r.	748–1297 mg GAE/100 g	[62]
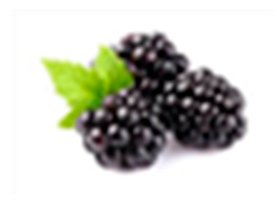 Blackberry (*Rubus fructicosus* L.)	n.r.	n.r.	2262–2395	[15,16,38,63]
	5.07–217 mg Ma3Gl/L	161–774 mg GAE/L	868–2581	
	n.r.	n.r.	759.42	
	n.r.	n.r.	4029 mg EAE/L	
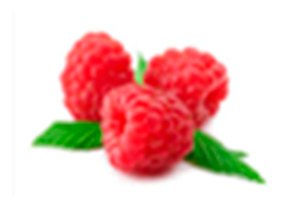 Raspberry (*Rubus idaeus*)	n.r.	n.r.	1441.61–1544.37	[15,38]
	n.r.	n.r.	566.75	
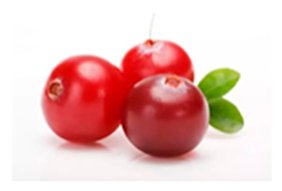 Cranberry (*Vaccinium* spp.)	n.r.	n.r.	408.03	[38]
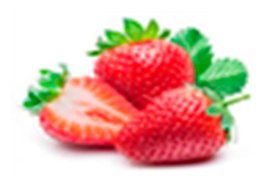 Strawberry (*Fragaria* × *ananassa*)	n.r.	n.r.	1438–1585.35	[19,38,64]
	n.r.	n.r.	525.6	
	n.r.	20.7–30.8 mg Pe3Gl/100 g	n.r.	
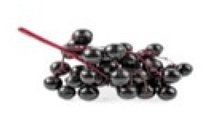 Elderberry (*Sambucus spp.*)	n.r.	n.r.	1480.47	[38]
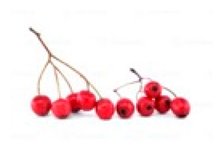 Hawthorn (*Crataegus* spp.)	n.r.	n.r.	1.91–2.44 mg GAE/mL	[65]
Pome fruit				
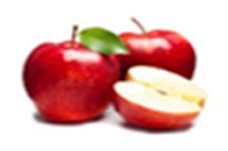 Apple (*Malus domestica* L.)	n.r.	n.r.	605–798.4	[15,51,65]
	n.r.	n.r.	228–639	
	n.r.	n.r.	0.48–0.82 mg GAE/mL	
Apple cider pomace	n.r.	n.r.	5.5–10.9 g GAE/kg	[44,66,67]
	n.r.	4672.5 mg/L	6.5 g GAE/100 kg	
	n.r.	n.r.	0.1 mg/mL	
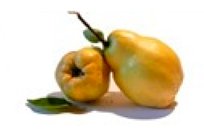 Japanese quince (*Chaenomeles japonica*) wine pomace	n.r.	n.r.	547–898 mg GAE/100 g	[62]
Drupe fruit				
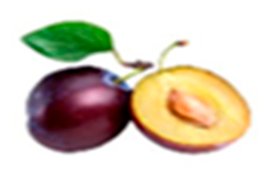 Plum (*Prunus domestica* L.)	n.r.	n.r.	1011.33–1389.48	[19,65]
	n.r.	n.r.	1.41–2.32 mg GAE/mL	
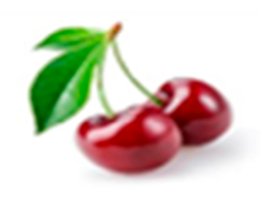 Sweet cherry (*Prunus avium* L.)	n.r.	n.r.	1586.31–1855.68	[19]
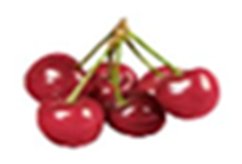 Sour cherry (*Prunus cerasus* L.)	n.r.	n.r.	1855.43–2281.52	[15]
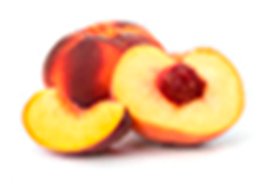 Peach (*Prunus persica* L.)	n.r.	n.r.	480.72–617.85	[19,20]
	n.r.	51.09–84.67 mg CAE/L	229.13–286.33	
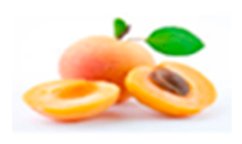 Apricot (*Prunus armeniaca* L.)	n.r.	n.r.	542.21–722.72	[19]
Multiple (Syconium) fruit				
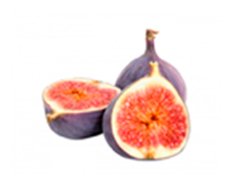 Fig (*Ficus carica*)	n.r.	92.32–110.61 mg RE/L	250.46–304.15	[68]
Tropical fruits				
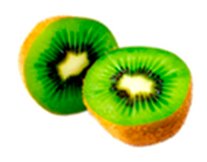 Kiwi (*Actinidia chinensis*)	n.r.	n.r.	390.42–2383.49	[56]
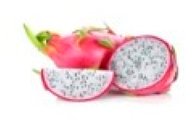 Dragon fruit (*Selenicereus undatus*)	n.r.	n.r.	280.61–387.30	[21]
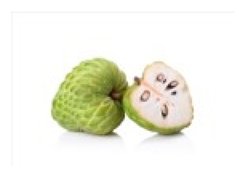 Custard apple (*Annona* spp.)	n.r.	n.r.	980	[22]
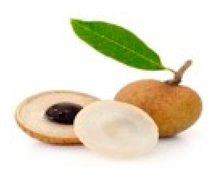 Longan (*Dimocarpus longan*)	n.r.	n.r.	2.34–5.35 mM/GE	[23]
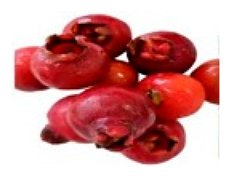 Red araça (*Psidium cattleyanum*)	7.5–8.5 mg Cy3Gl/L	n.r.	600–1000	[69]

n.r.—not recorded.

**Table 3:** Antiradical and antioxidant capacity of fruit wines and pomaces.

Fruit Wine	FRAP	ABTS	DPPH	ORAC	References
Berry Fruit					
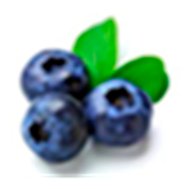 Blueberry (*Vaccinium myrtillus*)	70.11–87.78 mmol/L Fe^2+^	n.r.	n.r.	n.r.	[15,16,17,38]
	5.07 mM TE	n.r.	1.51–1.42%	n.r.	
	n.r.	4.29 mM TE	2.47 mM TE	n.r.	
	n.r.	1539.8 mM TE/L	1688 mM TE/L	25.3 mmol TE/L	
Blueberry wine pomace	n.r.	EC_50_ = 1.53 ± 0.09 mg/mL	EC_50_ = 2.16 ± 0.11 mg/mL	n.r.	[60]
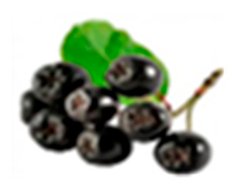 Black chokeberry (*Aronia melanocarpa*)	72.38–87.13 mmol/L Fe^2+^	n.r.	1.53–1.18%	n.r.	[15]
Black chokeberry wine pomace	n.r.	n.r.	422–617 mg AAE/100 g	n.r.	[62]
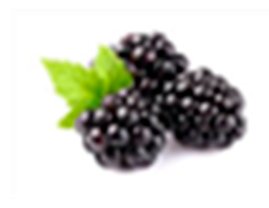 Blackberry (*Rubus fructicosus* L.)	97.17–115.23 mmol/L Fe^2+^	n.r.	5.25–4.18%	n.r.	[15,16,38,63]
	n.r.	1.23–7.77 mg/L	4.39–6.69 mg/L	n.r.	
	6.45 mM TE	5.84 mM TE	2.55 mM TE	n.r.	
	n.r.	n.r.	n.r.	37.4 mmol TE/L	
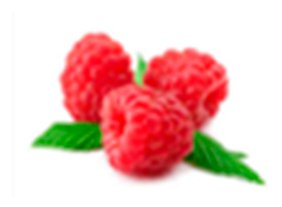 Raspberry (*Rubus idaeus*)	22.59–34.85 mmol/L Fe^2+^	n.r.	5.25–4.18%	n.r.	[15,38]
	4.42 mM TE	3.49 mM TE	1.75 mM TE	n.r.	
Raspberry wine pomace	0.631–1.162 mmol Fe^2+^/g	n.r.	24.1–79.0 µmol TE/g	n.r.	[71]
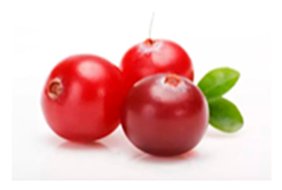 Cranberry (*Vaccinium* spp.)	3.32 mM TE	3.03 mM TE	1.12 mM TE	n.r.	[38]
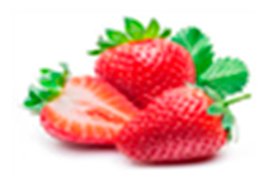 Strawberry (*Fragaria* × *ananassa*)	58.73–65.23 mmol/L Fe^2+^	n.r.	4.52–3.90%	n.r.	[19,38,64]
	3.47 mM TE	3.40 mM TE	1.66 mM TE	n.r.	
	50.3–71.2µmol Fe^2+^/100 g	56.5–69.1 µmolTE/100 g	n.r.	n.r.	
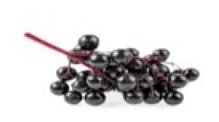 Elderberry (*Sambucus spp.*)	5.07 mM Trolox Eq	4.22 mM TE	1.87 mM TE	n.r.	[38]
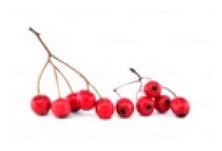 Hawthorn (*Crataegus* spp.)	n.r.	2.25–2.97%	0.97–1.24%	n.r.	[65]
Pome fruit					
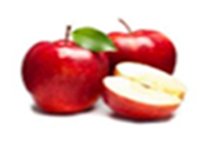 Apple (*Malus domestica* L.)	41.87–52.13 mmol/L Fe^2+^	n.r.	87.32–51.18%	n.r.	[15,50,65]
	n.r.	88.7–466.1 mgTE/100 mL	n.r.	n.r.	
	n.r.	0.45–0.68%	0.32–0.48%	n.r.	
Apple wine (cider) pomace	n.r.	n.r.	4.4–16.0 g AA/kg	n.r.	[44,66,67]
	1.02 mM/µL Fe^2+^	4.1–14.3 g AA/kg	245 g GAE/100 kg d.b.	n.r.	
	n.r.	n.r.	530.84 µm TEAC/µL	n.r.	
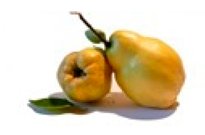 Japanese quince (*Chaenomeles japonica*) wine pomace	n.r.	n.r.	331–379 mg AAE/100 g	n.r.	[62]
Drupe fruit					
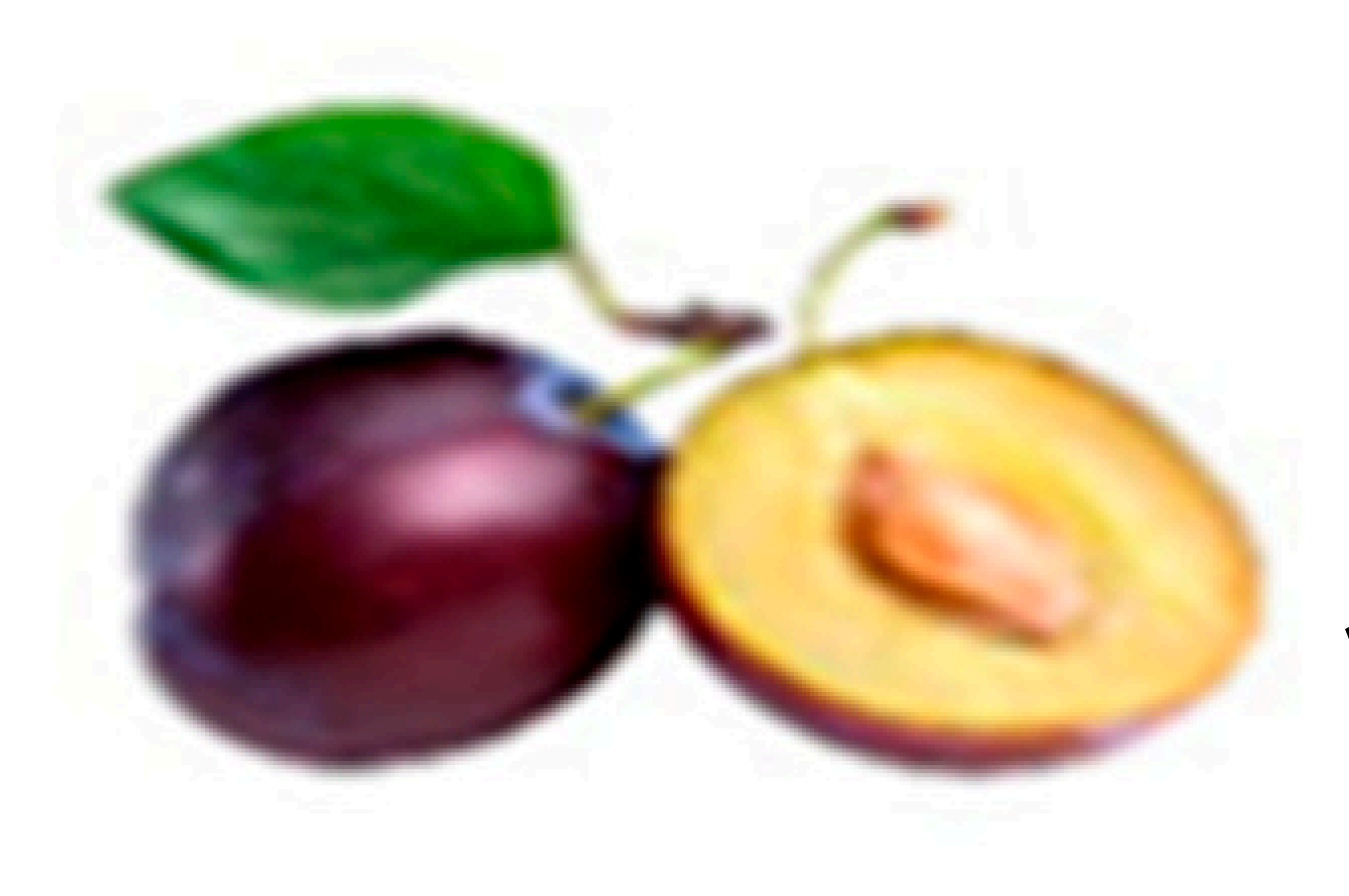 Plum (*Prunus domestica* L.)	52.75–61.95 mmol/L Fe^2+^	n.r.	42.13–27.55%	n.r.	[19,65]
	n.r.	1.35–1.73%	0.92–1.41%	n.r.	
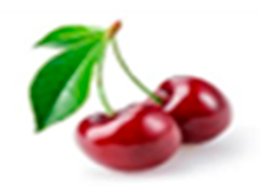 Sweet cherry (*Prunus avium* L.)	45.31–59.77 mmol/L Fe^2+^	n.r.	2.21–1.87%	n.r.	[19]
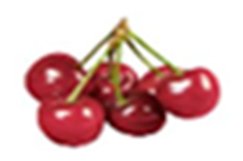 Sour cherry (*Prunus cerasus* L.)	50.33–64.89 mmol/L Fe^2+^	n.r.	1.97–1.59%	n.r.	[15]
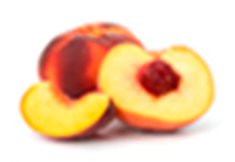 Peach (*Prunus persica* L.)	24.77–31.71 mmol/L Fe^2+^	n.r.	65.38–47.31%	n.r.	[19,20]
	~1.8–2.4 mM/L TE	~1.4–1.6 mM/L TE	~0.5–0.7 mM/L TE	n.r.	
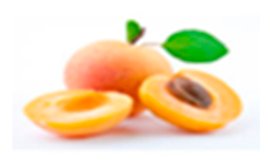 Apricot (*Prunus armeniaca* L.)	30.98–36.83 mmol/L Fe^2+^	n.r.	72.28–51.35%	n.r.	[19]
Multiple (syconium) fruit					
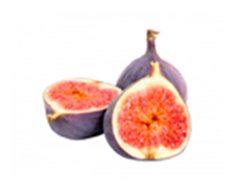 Fig (*Ficus carica*)	n.r.	n.r.	59.58–75.76%	n.r.	[68]
Citrus fruit					
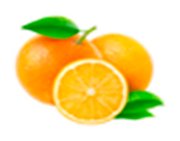 Orange (*Citrus* × *sinensis*)	n.r.	n.r.	0.46 mg/mL	n.r.	[18]
Tropical fruit					
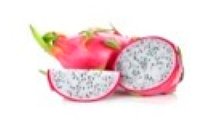 Dragon fruit (*Selenicereus undatus*)	n.r.	n.r.	n.r.	9.44–9.99 mM TE	[21]
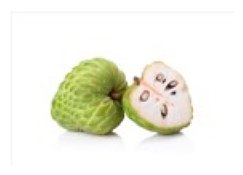 Custard apple (*Annona* spp.)	n.r.	n.r.	36.81–99.56%	n.r.	[22]
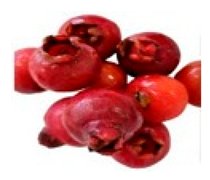 Red araçá (*Psidium cattleyanum*)	0.67–0.82 µM/mL TE	14.38–15.40 µM/mL TE	19.85–21 µM/mL TE	n.r.	[69]
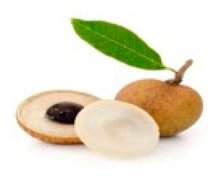 Longan (*Dimocarpus longan*)	0.85–11.21 mM FE	0.79–5.95 mM TE	n.r.	n.r.	[23]

n.r.—not recorded.

## Data Availability

No new data were created or analyzed in this study. Data sharing is not applicable to this article.

## Abbreviations

The following abbreviations are used in this manuscript:

TAC	Total Anthocyanin Content
TFC	Total Flavonoid Content
TPC	Total Phenolic Content
GAE	Gallic Acid Equivalent
FRAP	Ferric Reducing Antioxidant Power
ABTS	2,2′-Azino-bis(3-ethylbenzothiazoline-6-sulfonic acid
DPPH	2,2-Diphenyl-1-picrylhydrazyl
ORAC	Oxygen Radical Absorbance Capacity
TTA	Total Titratable Acidity
CAE	Caffeic Acid Equivalent
AAE	Ascorbic Acid Equivalent
AA	Ascorbic Acid
TEAC	Trolox Equivalent Antioxidant Capacity
RE	Retinol Equivalent
Cy3Gl	Cyanidin-3-Glucoside
C3GE	Cyanidin-3-Glucoside Equivalent
EAE	Ellagic Acid Equivalent
Ma3Gl	Malvidin-3-Glucoside
Pe3Gl	Peonidin-3-Glucoside
